# The Protein Encoded by the UL3.5 Gene of the Duck Plague Virus Affects Viral Secondary Envelopment, Release, and Cell-to-Cell Spread

**DOI:** 10.3390/vetsci12060510

**Published:** 2025-05-23

**Authors:** Huanhuan Cao, Bin Tian, Yanming Tian, Dongjie Cai, Mingshu Wang, Renyong Jia, Shun Chen, Anchun Cheng

**Affiliations:** 1 Engineering Research Center of Southwest Animal Disease Prevention and Control Technology, Ministry of Education of the People’s Republic of China, Chengdu 611130, China; fansdhn@126.com (H.C.); btian_1985@163.com (B.T.); tyanming2022@163.com (Y.T.); mshwang@163.com (M.W.); cqrc_jry@163.com (R.J.); shunchen@sicau.edu.cn (S.C.); 2 Research Center of Avian Disease and Institute of Veterinary Medicine and Immunology, College of Veterinary Medicine, Sichuan Agricultural University, Chengdu 611130, China; 3 Key Laboratory of Animal Disease and Human Health of Sichuan Province, Sichuan Agricultural University, Chengdu 611130, China; dongjie_cai@sicau.edu.cn; 4 State Key Laboratory of Green Pesticide, Institute of Veterinary Immunology and Green Drugs, Veterinary Department in College of Animal Science, Guizhou University, Guiyang 550025, China

**Keywords:** duck plague virus, UL3.5, secondary envelopment, virus release, cell-to-cell spread

## Abstract

Duck plague (DP), caused by duck plague virus (DPV), is a highly contagious and fatal disease among waterfowl. UL3.5, an unconserved gene belonging to the *Herpesviridae* family, *Alphaherpesvirinae* subfamily, and *Mardivirus* genus, is located downstream of UL3 and exhibits high variability in size and sequence, with an absence in herpes simplex virus (HSV). Currently, there is little understanding of DPV UL3.5. In this study, we determined that DPV pUL3.5 is distributed within the cytoplasm and co-located with multiple organelles. In addition, we investigated the genetic type of DPV UL3.5 and found that it is an early gene encoding an early viral protein. To further explore the function of DPV UL3.5, we constructed DPV-BAC-δUL3.5 and discovered that the deletion of UL3.5 significantly impacts the viral secondary envelopment and release processes. Furthermore, the UL3.5-deleted virus shows defects in cell-to-cell spread. In conclusion, our findings demonstrate, for the first time, that the early viral protein encoded by DPV UL3.5 plays a crucial role in promoting viral replication. This offers fundamental insights for further investigations into the function of DPV UL3.5.

## Simple Summary

Duck plague virus (DPV) severely threatens waterfowl, yet the role of its UL3.5 gene remains unknown. This study aimed to define UL3.5’s function by analyzing its protein localization, gene classification, and impact on viral replication. Results show that UL3.5 encodes an early cytoplasmic protein and is essential for viral secondary envelopment, release, and cell-to-cell spread, as its deletion cripples these processes. We conclude that UL3.5 is a key participant in the life cycle of the duck plague virus. These findings unveil a key viral replication mechanism and identify UL3.5 as a potential target for antiviral strategies, offering foundational information for further investigations into the functional characteristics of DPV UL3.5.

## 1. Introduction

Duck viral enteritis (DVE), commonly known as duck plague (DP), is a globally distributed disease that causes substantial economic losses in waterfowl breeding. The causative agent, duck plague virus (DPV), is classified within the *Herpesviridae* family, *Alphaherpesvirinae* subfamily, and *Mardivirus* genus [1,2,3,4]. DPV is a typical herpesvirus particle with a diameter of about 120–130 nm. Its structure consists of four parts, arranged from the outside to the inside: the envelope, tegument, capsid, and DNA genome core. The core contains linear double-stranded DNA that is entangled with protein. The genome of DPV (~160 kb) consists of four regions: a unique long region (UL), a unique short region (US), a terminal reverse repeat (TR), and an internal reverse repeat (IR) [5,6,7]. Transmission occurs via direct and indirect routes with high efficiency. The virus remains infectious in contaminated water (4–20 °C) for up to 60 days [8], and exhibits bidirectional transmission between domestic ducks and wild waterfowl [9].

The DPV life cycle follows the canonical herpesvirus stages: adsorption, invasion, replication, and release. After entering the host, the virus first attaches to the surface of the cell membrane and binds to receptors, then releases its nucleocapsid into the cytoplasm [10,11]. Subsequently, the DNA genome replicates in the nucleus and assembles with capsid proteins to form new nucleocapsids. These nucleocapsids then cross the nuclear membrane back into the cytoplasm for further maturation, undergoing two rounds of envelope wrapping during this process. Ultimately, the mature virions are released outside the cell through exocytosis [12,13,14,15]. DPV relies on its viral proteins to complete the entire life cycle. The genome of DPV contains 78 open reading frames (ORFs) encoding potential functional proteins, of which 65 ORFs are located in the UL region, 11 ORFs are located in the US region, and the remaining two (ICP4/IE180) are located in the IRS and TRS region. The proteins encoded by viral genes can be classified into structural proteins and non-structural proteins according to their functions. Structural proteins are essential for the formation and survival of virus particles. Non-structural proteins, also known as functional proteins, play multiple key roles in the viral life cycle, and their functions are involved in the entire process of viral invasion of the host, replication, assembly, and release [16,17,18].

Although members of the *Herpesviridae* family and *Alphaherpesvirinae* subfamily exhibit significant similarity in their DNA genome structure, with genes arranged collinearly across respective genomic regions, notable differences exist. In herpes simplex virus (HSV), varicella-zoster virus (VZV), equine herpesvirus-1 (EHV-1), pseudorabies virus (PrV), bovine herpesvirus-1 (BHV-1), and infectious laryngotracheitis virus (ILTV), UL region genes are sequentially named UL1–UL5 [19,20]. Except for HSV-1 and HSV-2, an additional ORF downstream of UL3 has been detected in the genomes of these viruses. This homolog is designated as the 57 gene in VZV, the 59 gene in EHV-1, and the UL3.5 gene in PrV, BHV-1, and ILTV [21,22,23,24,25,26]. Therefore, the UL3.5 homolog belongs to a very small number of genes that are not conserved throughout the *Alphaherpesvirinae* subfamily. Extensive studies on the PrV UL3.5 gene show it encodes a non-structural protein that may have a more stable interaction with subcellular compartments and is necessary for the secondary envelopment of intracellular virus particles across the Golgi apparatus [27].

The function of the UL3.5 gene may be closely linked to the pathogenicity of the above pathogens. When the UL3.5 protein is absent, the naked capsids of PrV accumulate near the Golgi apparatus or Golgi-derived vesicles. This aggregation blocks the release of viral particles [27]. However, inserting the BHV-1 UL3.5 homolog into UL3.5-deficient PrV restores viral replication and release. This functional rescue suggests that UL3.5 proteins from PrV and BHV-1 share conserved roles in facilitating α-herpesvirus extracellular release [28]. Structural studies reveal that BHV-1 pUL3.5 is an envelope protein. It interacts with the alpha-transducing factor (alphaBTIF) [29], and its C-terminal 40-amino-acid region is essential for promoting viral replication [30]. Furthermore, both PrV and BHV-1 pUL3.5 directly bind to pUL48. This interaction is critical for viral particle assembly and neurovirulence [31]. Interestingly, the UL3.5 gene is absent in HSV-1 and HSV-2. Despite this absence, these viruses can still produce extracellular particles. This observation implies that other HSV-1 proteins may compensate for the function of pUL3.5. Potential candidates include pUL48 itself or envelope glycoproteins (gB, gD, and gH), which are known to interact with pUL48 [32,33]. Unlike PrV UL3.5 deletion, deletion of the VZV ORF57 homolog has no significant impact on the virus [34]. As a highly pathogenic agent like PrV, DPV retains the UL3.5 gene, which hints that this gene may be functionally relevant but currently uncharacterized.

The DPV UL3.5 encodes a putative 120-amino-acid protein with a molecular weight of ~13.4 kDa. This gene occupies a conserved genomic position between UL3 and UL4 in DPV, analogous to its homologs in BHV-1, PRV, and EHV-1; however, amino acid sequence alignment reveals only limited homology among their encoded proteins. Current research on UL3.5 has largely focused on mammalian herpesvirus (e.g., PrV), whereas its expression patterns, subcellular localization, and roles in avian herpesvirus (e.g., DPV) replication remain uncharacterized. This study systematically investigates DPV UL3.5 protein features and functional impacts on the viral life cycle to establish foundational data for understanding its biological roles. Using a bacterial artificial chromosome (BAC) platform, we successfully constructed and rescued recombinant virus strains DPV-BAC-NanoLuc-UL3.5, DPV-BAC-δUL3.5, and DPV-BAC-RUL3.5 via the Red recombination system. Findings reveal that DPV UL3.5 encodes an early protein critical for viral secondary envelopment, release, and cell-to-cell spread.

## 2. Materials and Methods

### 2.1. Cells, Viruses, and Primers

This study used duck embryo fibroblasts (DEFs), which were isolated from 9-day-old duck embryos (purchased from Ya’an City, China) and cultured in Dulbecco’s Modified Eagle Medium (DMEM, Servicebio, Wuhan, China) supplemented with 10% fetal bovine serum (FBS, YEASEN, Shanghai, China) and 1% penicillin–streptomycin (Solarbio, Beijing, China). Cells were maintained at 37 °C in 5% CO_2_. The virus used was a DPV-BAC recombinant virus strain containing an EGFP selective marker gene, generated via a BAC-based system from the virulent Chinese strain of duck enteritis virus (DEV CHv, GeneBank: JQ647509.1) isolated and preserved in our laboratory. The complete sequences of all primers utilized in this study are presented in Appendix A Table A1.

### 2.2. Antibodies

Rabbit polyclonal antibodies against ICP27, UL28, and UL47 were prepared and preserved by the Research Center of Avian Disease, Sichuan Agricultural University. Mouse anti-NanoLuc antibody (1:1000) was supplied by Promega Biosciences (Madison, WI, USA). Mouse anti-GFP antibody (1:1000) was obtained from Beyotime Biotechnology (Shanghai, China). Mouse anti-GAPDH antibody (1:5000) was provided by Abmart (Shanghai, China). HRP-conjugated goat anti-rabbit and goat anti-mouse IgG (1:5000) were purchased from Proteintech (Wuhan, China).

### 2.3. Construction of Plasmid and Recombinant BAC Virus Plasmid

The DPV UL3.5 gene sequence (Gene ID: 80532517) was cloned into the pEGFP-N1 expression vector, hereafter referred to as pEGFP-N1-UL3.5. The pDsRed2-ER and pDsRed2-Mito plasmids were maintained in our laboratory. A bacterial artificial chromosome (BAC) is a bacterial chromosomal cloning vector based on the F-plasmid of Escherichia coli [35]. Our laboratory has constructed and obtained a GS1783 Escherichia coli containing the complete genome sequence of DPV CHv. On this basis, DPV-BAC-NanoLuc-UL3.5, DPV-BAC-δUL3.5, and DPV-BAC-RUL3.5 were constructed by a two-step marker-free Red recombination technology [36]. Primers containing homologous arms were designed to amplify the linear target gene product, which carried the KanR cassette. The first Red recombination replaced the UL3.5 gene fragment, and the KanR cassette was deleted in the second Red recombination by cleaving the I-SecI recognition site. DPV-BAC-NanoLuc-UL3.5 and DPV-BAC-RUL3.5 were generated using the same methodology.

### 2.4. Conventional Polymerase Chain Reaction (PCR)

The reaction mixture was prepared on ice, containing the following components: appropriate amounts of DNA template (viral DNA at a final concentration of 10 ng/μL, cDNA at 50 ng/μL), 0.5 μL each of forward and reverse primers (final concentration 0.5 μM), 5 μL of 2× Hieff Canace**^®^** Gold PCR Master Mix (YEASEN, Shanghai, China), and additional ddH_2_O for a final volume of 10 μL. Reaction tubes were subsequently placed in a thermal cycler programmed with the following parameters: pre-denaturation at 98 °C for 3 min (1 cycle); denaturation at 98 °C for 10 s, annealing at 55 °C for 15 s, and extension at 72 °C for 10 s/kb (35 cycles); and a final extension at 72 °C for 10 min. The PCR products were analyzed with agarose gel electrophoresis using a 1% agarose gel containing 0.5 μg/mL ethidium bromide. Electrophoresis was performed at 120 V/cm for 15–20 min, with a DNA ladder serving as the molecular weight reference. Target bands were visualized under a UV imaging system (Bia-Rad, Hercules, CA, USA).

### 2.5. Rescue and Identification of Recombinant Virus

The positive BAC plasmid was extracted using the NucleoBond Xtra Midi kit (MACHEREY-NAGEL, Düren, Germany) and subsequently transfected into DEFs with HiefTrans^TM^ Liposomal Transfection Reagent (YEASEN, Shanghai, China). Following transfection, cells were incubated at 37 °C in 5% CO_2_ for 6–8 h, after which the supernatant was discarded and cell surfaces washed with PBS. DMEM containing 2% FBS and 1% penicillin–streptomycin was then added, and the cells were further cultured until green fluorescent spots emerged. The supernatant was then collected to infect fresh DEF cells, with this viral passage and rescue process repeated for three generations. Viral DNA was extracted and subsequently utilized as the template for PCR-based identification of the rescued virus. Detailed reaction conditions and thermal cycling parameters are provided in Section 2.4.

### 2.6. Fluorescent Protein Labeling

DEFs were seeded into 12-well plates with glass coverslips, and transfected at appropriate density. Each well received 1 μg of plasmid DNA complexed with 2.5 μL of Hieff Trans^TM^ Liposomal Transfection Reagent (YEASEN, Shanghai, China). This study employed fluorescent marker proteins, enhanced green fluorescent protein (EGFP), and red fluorescent protein (DsRed2), to specifically label the target protein UL3.5 and subcellular organelles, respectively. At 36 h post-transfection, the cells were fixed with 4% paraformaldehyde (PFA) for 20 min at room temperature. The fixative solution was then discarded, and the cells were washed three times with PBST. Cellular membranes were permeabilized with 0.25% Triton X-100 for 10 min, followed by three PBST washes. Nuclei were stained with DAPI for 5 min. After three final PBST washes, specimens were mounted using ProLong™ Gold Antifade Mountant (Thermo Fisher Scientific, Waltham, MA, USA) under coverslips. Imaging was performed using a Nikon H550L fluorescent microscope (Nikon, Tokyo, Japan) equipped with a DS-Qi2 monochromatic camera (Nikon, Tokyo, Japan) and NIS-Elements AR 5.21 software under a 40× objective magnification.

### 2.7. Real-Time Quantitative PCR

Following infection of DEFs with DPV-BAC, cells were collected at different time points. Total RNA was extracted using TRIzol reagent (Invitrogen, Carlsbad, CA, USA). RNA concentration and purity (A260/A280 ratio of 1.8–2.0) were quantified spectrophotometrically, with all samples normalized to 500 ng. The RNA was then reverse-transcribed into cDNA using a First-strand cDNA Synthesis Mix (LABLEAD, Beijing, China). The real-time quantitative PCR reaction system was as follows: 0.5 μL each of gene-specific forward/reverse primer (sequences in Table A1), 5 μL of 2× Taq SYBR^®^ Green qPCR Premix (LABLEAD, Beijing, China), 1 μg of cDNA, and nuclease-free water to 10 μL. Thermal cycling protocol was performed as follows: 95 °C for 30 s, followed by 40 cycles at 95 °C for 10 s, 60 °C for 10 s, and 72 °C for 30 s. Transcriptional kinetics of UL3.5 were analyzed, with ICP27, UL28, and UL47 serving as controls for immediate early (IE), early (E), and late (L) genes [37]. Due to the constitutive stability of β-actin in eukaryotic cellular systems [38,39], duck β-actin was used as an internal reference gene to normalize the expression values of the target gene [40]. Each group contained three biological replicates, and relative transcription levels were calculated using the 2^−ΔΔCt^ method.

### 2.8. Cell Viability Assessment by CCK-8

Cell viability assessment was performed using the Cell Counting Kit-8 (CCK-8, Beyotime, C0038, Shanghai, China) to rule out nonspecific cytotoxicity induced by ganciclovir (GCV) or cycloheximide (CHX) at their maximum working concentrations. DEFs were seeded in 96-well plates at 100 μL/well and treated with GCV (300 μg/mL) or CHX (200 μg/mL) for 24 h. A dimethyl sulfoxide (DMSO)-treated group served as the vehicle control and a culture medium-only group as the blank control. Subsequently, 10 μL of CCK-8 reagent was added to each well, followed by incubation at 37 °C in the dark for 2 h. Absorbance at 450 nm was measured using a Varioskan LUX microplate reader (Thermo Fisher Scientific, USA). Cell viability (%) = (OD treatment) − (OD blank)/(OD control) − (OD blank) × 100.

### 2.9. GCV/CHX Drug Inhibition Assay

DEFs were infected with DPV-BAC at 0.1 MOI and simultaneously treated with 300 μg/mL of ganciclovir (GCV, a nucleic acid synthesis inhibitor; TargetMol, Wellesley Hills, MA, USA) or 200 μg/mL of cycloheximide (CHX, a protein synthesis inhibitor; MCE, Junction, NJ, USA). Mock-infected cells served as negative controls, and DMSO-treated virus-infected cells as vehicle controls. After 2 h of incubation at 37 °C in 5% CO_2_, the supernatant was removed, and the cells were washed with PBS three times. Growth maintenance medium containing GCV/CHX was added, and cells were further cultured for 24 h. Total cellular RNA was extracted, reverse-transcribed into cDNA, and used as the template. Gene-specific primers targeting UL3.5, ICP27, UL28, UL47, and β-actin (as an endogenous control) were added into the reaction system. PCR amplification was conducted using high-fidelity DNA polymerase (Vazyme, Nanjing, China). The resulting products were separated by 1% agarose gel electrophoresis to assess the effects of GCV and CHX on viral gene expression.

### 2.10. Western Blotting Analysis

DEFs were seeded in 12-well plates and cultured for 12–16 h until the cell density reached 80–90%, then infected with 0.5 MOI of recombinant virus DPV-BAC-NanoLuc-UL3.5 and incubated at 37 °C for 2 h. Following three PBS washes, the cells were maintained in DMEM supplemented with 2% FBS and 1% penicillin–streptomycin. Cellular specimens were harvested at defined post-infection intervals (0, 2, 4, 8, 12, 24, 36, 48, and 60 hpi). Total protein was extracted using SDS lysis buffer (Beyotime, P0013G, Shanghai, China) containing protease inhibitor cocktail (Merck, P8340, Darmstadt, Germany). The samples were boiled, separated by SDS-PAGE (Sodium Dodecyl Sulfate Polyacrylamide Gel Electrophoresis), and transferred onto Polyvinylidene Fluoride (PVDF) membranes (Beyotime, FFP39, Shanghai, China). The PVDF membranes were then blocked for 3 h at room temperature with a blocking buffer (PBS containing 5% skimmed milk powder and 0.1% Tween 20), then incubated overnight at 4 °C with primary antibodies (mouse anti-NanoLuc, rabbit anti-ICP27, rabbit anti-UL28, rabbit anti-UL47, and mouse anti-GAPDH). After washing, the membranes were incubated with secondary antibodies for 1 h at room temperature. Protein bands were visualized using an enhanced chemiluminescence (ECL) reagent (YEASEN, Shanghai, China).

### 2.11. Determination of Multi-Step Growth Curve In Vitro

DEFs were infected with 0.01 MOI of recombinant virus DPV-BAC, DPV-BAC-NanoLuc-UL3.5, DPV-BAC-δUL3.5, or DPV-BAC-RUL3.5. After incubation at 37 °C for 2 h, unbound virions were removed through three PBS washes. Following replacement with maintenance medium, the cells continued to be cultured at 37 °C. Supernatants and cell samples were collected at 24, 48, 72, and 96 hpi. Virus titers were determined to assess virus growth via 50% tissue culture infectious dose (TCID_50_) assays.

### 2.12. Virus Adsorption, Invasion, Replication, and Release Assays

#### 2.12.1. Adsorption

DEFs were pre-cooled at 4 °C for 1 h, and then infected with DPV-BAC, DPV-BAC-δUL3.5, and DPV-BAC-RUL3.5 at 0.1 MOI. Following incubation at 4 °C for 2 h, viral DNA was extracted using Magen HiPure Viral DNA Kit (Magen, Guangzhou, China). The viral copy number was determined using UL30 quantitative primers previously designed by our laboratory. There were three biological replicates in each group.

#### 2.12.2. Invasion

Virus infection of DEFs was performed as described in Section 2.12.1. After incubation at 4 °C for 2 h, the cells were washed three times with cold PBS. Maintenance medium was added, and cells were cultured at 37 °C for 3 h. Cell samples were collected for virus DNA extraction and virus copy number was determined.

#### 2.12.3. Replication

DEFs were infected with 0.1 MOI of viruses and incubated at 37 °C for 6 h, followed by replacement with 2% FBS growth maintenance medium. Viral DNA was extracted at 7, 8, 9, and 10 hpi for quantification of viral copy number.

#### 2.12.4. Release

DEFs were infected with viruses at an MOI of 1 and incubated under stationary conditions at 37 °C for 18 h. Following three PBS washes, DMEM containing 2% FBS was added. Supernatants were collected at 30, 60, 90, and 120 min post-medium replacement for TCID_50_ assays.

### 2.13. Electron Microscopy

DEFs were seeded in 60 mm culture dishes and allowed to reach 80% confluency. Cells were infected with recombinant viruses DPV-BAC, DPV-BAC-δUL3.5, and DPV-BAC-RUL3.5 at an MOI of 2. At 14 hpi, cells were detached by trypsinization (Servicebio, G4021-100ML, Wuhan, China) for 2 min. After centrifugation at 1000 rpm for 2 min, the supernatant was discarded. Cell pellets were fixed with 0.5% glutaraldehyde (diluted with PBS 1:6), followed by 5 min of incubation at 4 °C. Samples were centrifuged at 12,000 rpm for 10 min. The supernatant was carefully discarded, and the pellet was retained. A 3% glutaraldehyde fixative was slowly added along the tube wall. Processed samples were submitted to LILAI Medical Laboratory Center for electron microscopy analysis.

### 2.14. Analysis of Virus Cell-to-Cell Spread

DEFs were infected with DPV-BAC, DPV-BAC-δUL3.5, and DPV-BAC-RUL3.5 (0.001 MOI) at 37 °C for 2 h. Following three PBS washes, the cells were overlaid with a mixture of 2× DMEM containing 2% FBS and 2% agarose. After further incubation at 37 °C for 36 h, viral fluorescent plaques were observed under a fluorescence microscope. Then, 30 fluorescent spots were randomly selected, and the plaque areas were quantified using ImageJ software (NIH, Bethesda, MD, USA). The average plaque area of the DPV-BAC group was set as 100% and compared with that of DPV-BAC-δUL 3.5 and DPV-BAC-RUL3.5.

### 2.15. Data Analysis

Statistical analysis was performed using ImageJ/Fiji 1.53 (NIH, Bethesda, MD, USA) and GraphPad Prism version 9 (San Diego, CA, USA). One-way ANOVA was applied for comparisons involving multiple groups, while Student’s *t*-test was utilized for comparisons between two independent groups. Statistical significance was denoted as follows: *p* > 0.05 (ns), *p* ≤ 0.05 (*), *p* ≤ 0.01 (**), *p* ≤ 0.001 (***), *p* ≤ 0.0001 (****). Data are presented as mean ± SEM.

## 3. Results

### 3.1. DPV pUL3.5 Is a Multicellular Localization Protein

**Figure 1:**
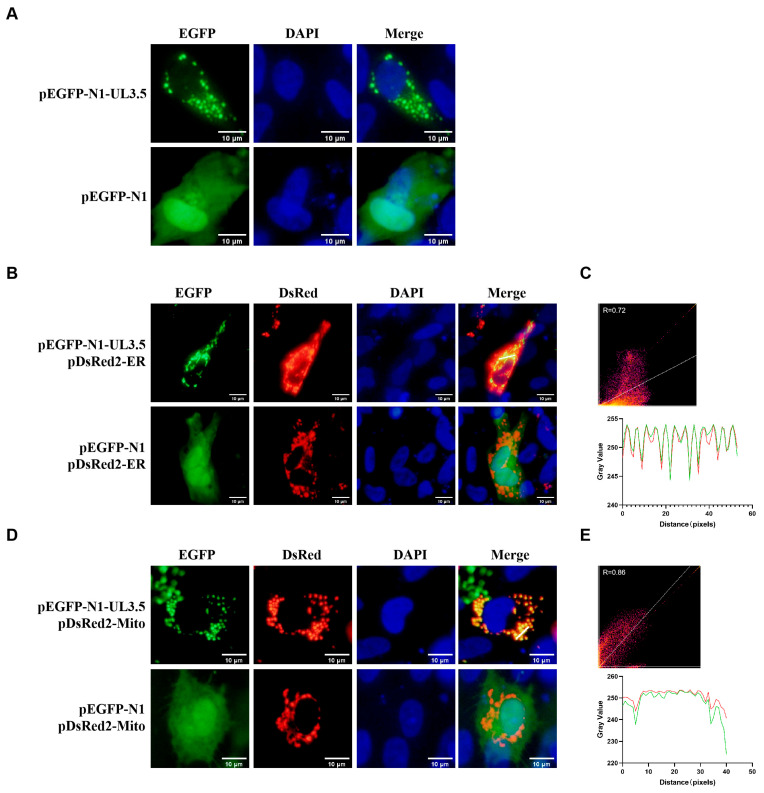
DPV pUL3.5 is distributed in cytoplasm and co-localized with mitochondria and ER (original figures see Supplementary Materials). (**A**): DPV pUL3.5 is distributed in cytoplasm. DEFs were transfected with pEGFP-N1-UL3.5 or pEGFP-N1. Dye the nucleus with DAPI. Scale bar: 10 μm. (**B**): DPV pUL3.5 co-localizes with ER. pEGFP-N1-UL3.5 was co-transfected with pDsRed2-ER into DEFs, with pEGFP-N1 serving as a vector control. DPV pUL3.5 is visualized in green, the ER in red, and the nuclei are stained with DAPI (blue). Scale bar: 10 μm. (**C**): Quantitative co-localization analysis between DPV pUL3.5 and the ER was performed using Pearson’s correlation coefficient (PCC)-based scatter plots and a fluorescence waveform analysis diagram of protein expression. The green line represents EGFP, and the red line represents DsRed2. Analysis was performed using ImageJ/Fiji 1.53 and GraphPad Prism version 9 software. (**D**): DPV pUL3.5 co-localizes with mitochondria. DEFs were transfected with pDsRed2-Mito along with either pEGFP-N1-UL3.5 or pEGFP-N1, where pEGFP-N1 served as the vector control. EGFP emits green light, representing DPV pUL3.5; DsRed2 emits red light, representing mitochondria; and the nuclei are stained with DAPI (blue). Scale bar: 10 μm. (**E**): Quantitative co-localization analysis between DPV pUL3.5 and mitochondria was performed using Pearson’s correlation coefficient (PCC)-based scatter plots and a fluorescence waveform analysis diagram of protein expression. The green line represents EGFP, and the red line represents DsRed2. Analysis was performed using ImageJ and GraphPad Prism version 9 software.

To determine the distribution of the protein encoded by DPV UL3.5 in host cells, we constructed an EGFP-tagged UL3.5 fusion plasmid (pEGFP-N1-UL3.5), and transfected it into DEF cells. DPV pUL3.5 was labeled with the product of pEGFP-N1-UL3.5 plasmid that emits green fluorescence. Additionally, pEGFP-N1 was transfected into DEFs as the vector control. DPV pUL3.5 exhibits a punctate distribution and is extensively localized in the cytoplasm; it is absent from a DAPI-stained cell nucleus (Figure 1A). Then, we further explored the specific subcellular localization of pUL3.5. The pEGFP-N1-UL3.5 plasmid was co-transfected with the pDsRed2-ER or pDsRed2-Mito plasmid; meanwhile, cells co-transfected with pEGFP-N1 and either pDsRed2-ER or pDsRed2-Mito plasmids were established as vector controls. The endoplasmic reticulum (ER) and mitochondria were labeled with the products of the pDsRed2-ER and pDsRed2-Mito plasmid, respectively, which emitted red fluorescence, and pUL3.5 was labeled with green fluorescence. It was found that pUL3.5 is co-located with the ER (Figure 1B) and mitochondria (Figure 1D). Quantitative co-localization analysis using Pearson’s correlation coefficient (PCC) revealed significant positive correlations between pUL3.5 and subcellular organelles: Rcoloc = 0.72 with ER (Figure 1C) and Rcoloc = 0.86 with mitochondria (Figure 1E). Localized fluorescence intensity profiling demonstrated synchronized pixel-level covariation between pUL3.5 and organellar markers (Figure 1C,E). These data show that DPV pUL3.5 is a multi-subcellular viral protein co-located with the ER and mitochondria.

### 3.2. DPV UL3.5 Encodes an Early Viral Protein

**Figure 2:**
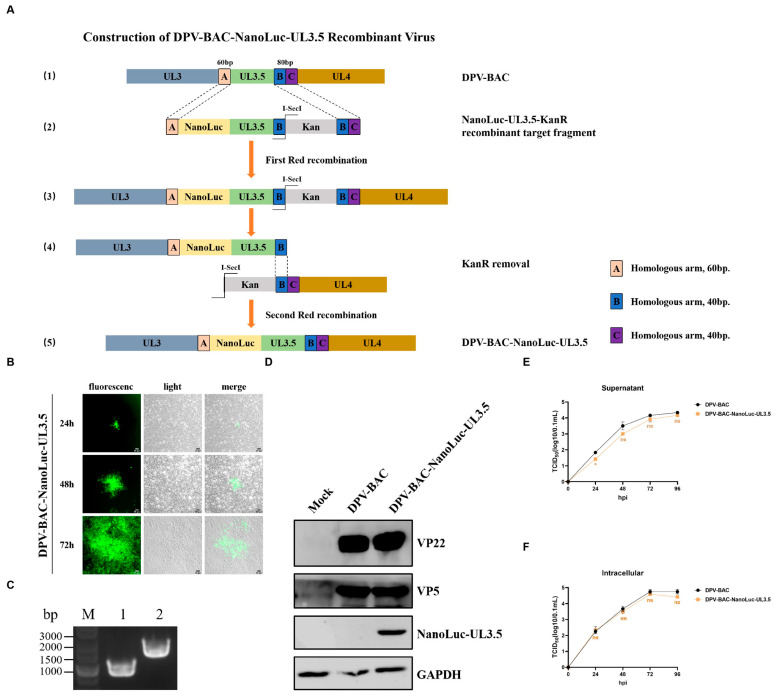
Construction and identification of DPV-BAC-NanoLuc-UL3.5 recombinant virus (original figures see Supplementary Materials). (**A**): Schematic diagram of the genome structure of DPV-BAC-NanoLuc-UL3.5. (1) The gene structure of UL3, UL3.5, and UL4 in DPV-BAC. Fragments A, B, and C are homologous arms at both ends of UL3.5; homologous arm A is 60 bp and homologous arms B and C are 40 bp. (2) NanoLuc, UL3.5, and KanR cassette sequences were amplified with primers, and the three sequences were fused into a single DNA fragment via two fusions. The first Red recombination replaced the UL3.5 gene with the NanoLuc-UL3.5-KanR recombinant target fragment through homologous arm A and homologous arm B + C. (3) The product after the first Red recombination. (4) The homing endonuclease I-SecI removed the Kana fragment, and the second Red recombination was completed through homologous arm B. (5) Structure diagram of DPV-BAC-Nanoluc-UL3.5. (**B**): Rescue of the DPV-BAC-NanoLuc-UL3.5 recombinant strain. Infectious clone plasmids were extracted and transfected into DEFs for virus rescue. Viral fluorescent plaques were observed 24 h later, and the EGFP fluorescent protein of the recombinant virus continued to express over time. Scale bar: 50 μm. (**C**): PCR identification of DPV-BAC-NanoLuc-UL3.5. Viral DNA was extracted from cells infected with DPV-BAC-NanoLuc-UL3.5 and DPV-BAC, and the UL3.5 gene was amplified using identification primers. Lane 1: Using DPV-BAC DNA as the template. Lane 2: Using DPV-BAC-NanoLuc-UL3.5 DNA as the template. (**D**) WB identification of DPV-BAC-NanoLuc-UL3.5. DEFs were infected with 1 MOI of DPV-BAC-NanoLuc-UL3.5, mock-infected cells maintained in complete DMEM were established as negative control for infection, parallel experimental group infected with DPV-BAC was designated as wild-type control. Cells then were collected at 24 hpi, and proteins were extracted by using SDS lysis buffer for WB analysis. VP22 and VP5 were used as infection controls, and GAPDH served as internal reference control for the experiment. (**E**,**F**) Multi-step growth curve assay of BAC-DPV-NanoLuc-UL3.5 and DPV-BAC. DEFs were infected with 0.1 MOI of viruses, and the supernatants and cell samples were harvested at 24, 48, 72, and 96 hpi for TCID_50_ determination. Data from each group represent three independent experiments. The dispersion analysis was presented as mean ± SEM. The *t*-test was used to analyze the statistical difference in virus titers between BAC-DPV-NanoLuc-UL3.5 and DPV-BAC (ns > 0.05, * *p* ≤ 0.05).

To characterize the DPV UL3.5 gene, we constructed a DPV-BAC-NanoLuc-UL3.5 luciferase recombinant virus, as there is no antibody against the DPV UL3.5-encoded protein. Using primers listed in Table A1, we employed a two-step marker-free Red recombination strategy on the BAC platform of the Chinese virulent DEV CHv strain to generate an infectious clone plasmid carrying NanoLuc luciferase (DPV-BAC-NanoLuc-UL3.5). The main construction process is shown in Figure 2A. Following sequence verification, the positive infectious clone plasmid was transfected into DEFs for virus rescue. The fluorescent plaque and cellular morphological changes within 72 h are shown in Figure 2B. Then, we identified the DPV-BAC-NanoLuc-UL3.5 virus via PCR and Western Blotting (WB) analysis. As shown in Figure 2C, the band amplified using the NanoLuc-UL3.5 fragment from DPV-BAC-NanoLuc-UL3.5 as a template has a size of 1574 bp (lane 2), while the DNA band amplified from the UL3.5 sequence of DPV-BAC is 953 bp (lane 1). The results demonstrate that the NanoLuc luciferase reporter gene was successfully inserted into the viral genome and fused to the UL3.5 gene. Virus-infected cell samples were collected for WB analysis. Mock-infected cells treated with complete DMEM medium were established as negative infection control, and cells infected with DPV-BAC served as wild-type control. NanoLuc-UL3.5 could be detected in the recombinant virus DPV-BAC-NanoLuc-UL3.5. VP5 and VP22 were used as the virus infection control, and GADPH was used as the internal reference control (Figure 2D). Growth kinetics assays revealed no significant differences in virus titers between DPV-BAC and DPV-BAC-NanoLuc-UL3.5 (Figure 2E,F), indicating that NanoLuc luciferase does not impair viral replication. Thus, some of the subsequent experiments can use this strain instead of DPV-BAC.

**Figure 3:**
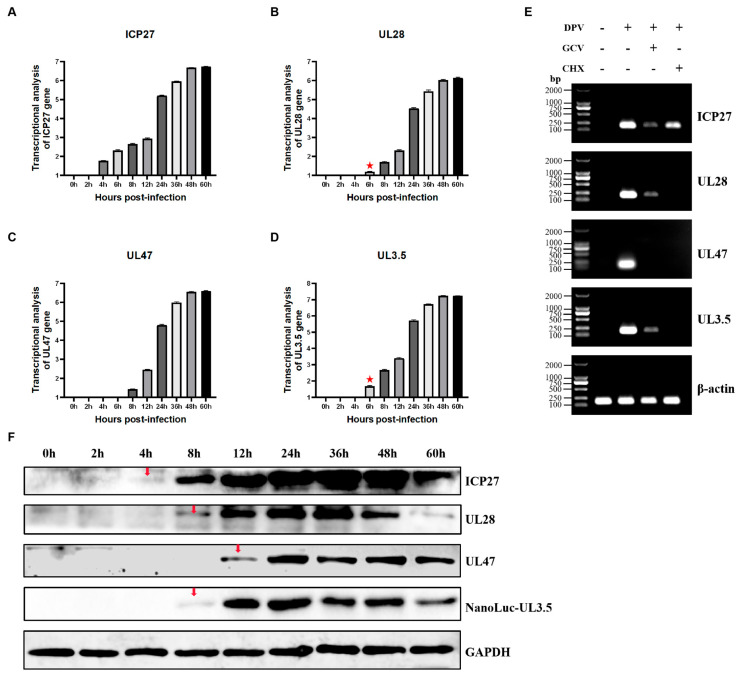
DPV pUL3.5 is an early viral protein (original figures see Supplementary Materials). (**A**–**D**): DEFs were infected with 0.1 MOI of DPV-BAC. Cell samples were collected at 0, 2, 4, 6, 8, 12, 24, 36, 48, and 60 hpi for total RNA extraction, and the cDNA obtained by reverse-transcription was used as the template for RT-qPCR. Duck β-actin was designated as the internal reference gene to detect the relative mRNA levels of UL3.5, ICP27, UL28, and UL47. ICP27, UL28, and UL47 served as control for IE, E, and L genes, respectively. Red asterisks indicate that the transcriptional levels of both UL28 and UL3.5 began to increase from 6 hpi. (**E**): DEFs were infected with DPV-BAC at 0.1 MOI, and simultaneously treated with GCV/CHX. DMSO-treated virus-infected cells served as vehicle controls, and mock-infected DEFs were established as negative controls. Total RNA was extracted at 24 hpi, reverse-transcribed into cDNA, and subjected to conventional PCR amplification. Amplification products were analyzed via 1% agarose gel electrophoresis. The characteristics of the UL3.5 gene were identified by assessing the impact of GCV and CHX on the expression of various genes. (**F**): DEFs were infected with 0.5 MOI of DPV-BAC-NanoLuc-UL3.5, and the cells were collected at 0, 2, 4, 8, 12, 24, 36, 48, and 60 hpi for Western Blotting. The ICP27, UL28, and UL47 proteins, respectively, represent IE, E, and L proteins, while GAPDH served as the internal reference protein. Red arrowheads denote the initial detection time points of individual viral proteins in the corresponding panels.

To classify the protein type of DPV pUL3.5, we first analyzed the transcriptional kinetics of the UL3.5 gene using real-time quantitative PCR (RT-qPCR). RT-qPCR was employed to analyze transcriptional changes of target genes. Owing to its exceptional sensitivity in detecting RNA/DNA at ultralow copy numbers (as low as single-digit copies), this method enables the capture of early transcriptional signatures of viral genes even under low viral loads. DEFs were infected with 0.1 MOI of DPV-BAC, the cells were harvested at various hours post-infection, and RNA was extracted and reverse-transcribed into cDNA for RT-qPCR analysis of UL3.5 mRNA levels. ICP27, UL28, and UL47 served as controls of IE, E, and L genes, respectively, with β-actin as the reference gene. Results showed that ICP27 transcription increased from 4 hpi, UL28 from 6 hpi, UL47 from 8 hpi, and UL3.5 exhibited a pattern similar to UL28, rising from 6 hpi (Figure 3A–D). To further validate these findings, GCV/CHX treatment assays were performed. Cell viability post-treatment was assessed using the CCK-8 kit to confirm the absence of cytotoxic effects from GCV or CHX at the applied concentrations (Table A2). DEFs were infected with DPV and treated with GCV/ CHX, DMSO-treated virus-infected cells served as vehicle controls, and mock-infected DEFs were established as negative controls. RNA was extracted, reverse-transcribed into cDNA, and subjected to PCR analysis. The results indicate that GCV did not affect the expression of DPV UL3.5, whereas CHX treatment stopped its expression, which was the same as that of the early gene UL28 (Figure 3E). Then, we examined the protein kinetics of DPV pUL3.5. DEFs were infected with 0.5 MOI of DPV-BAC-NanoLuc-UL3.5, and cell samples were obtained at various hours post-infection for WB analysis. Western Blotting, which relies on antibody–antigen binding specificity, typically requires higher protein expression levels to generate detectable signals, particularly for low-abundance proteins. To address this limitation, a higher multiplicity of infection was selected for viral infection compared to the MOI employed in qPCR-based transcriptional phase analyses. The imaging results showed that weak protein blotting of ICP27 was detected at 4 hpi, protein expression of UL3.5 and UL28 was detected at 8 hpi, while UL47 began to express at 12 hpi (Figure 3F). Therefore, the UL3.5 protein shares a similar protein expression kinetic pattern with the early protein UL28. The above results suggest that DPV pUL3.5 is an early viral protein.

### 3.3. Construction and Rescue of DPV-BAC-δUL3.5 and DPV-BAC-RUL3.5 Recombinant Virus Strains

**Figure 4:**
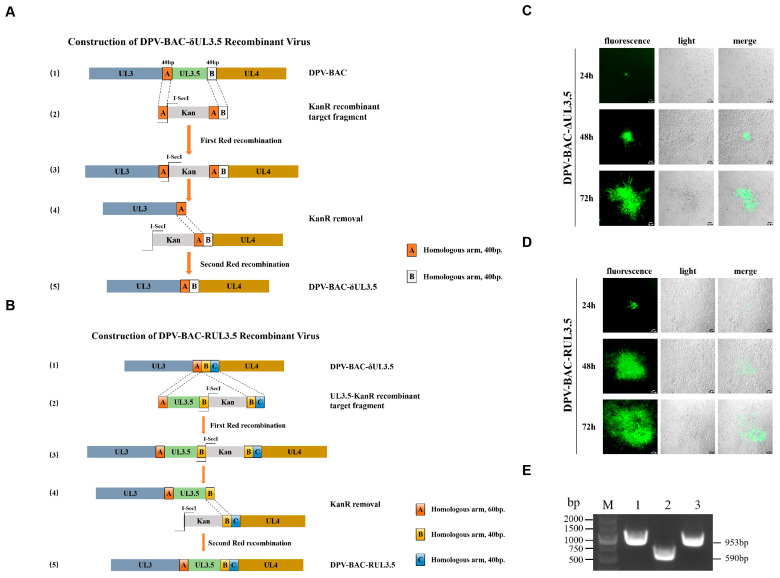
Construction and rescue of the DPV-BAC-δUL3.5 deletion mutant and the DPV-BAC-RUL3.5 reverted virus strains (original figures see Supplementary Materials). (**A**): Schematic diagram of the genome structure of DPV-BAC-δUL3.5. (1) Schematic of the gene structures of UL3, UL3.5, and UL4 in DPV-BAC. A and B denote the homologous arms at both ends of UL3.5, each 40 bp in length. (2) The KanR cassette sequence was amplified, and the first recombination replaced the UL3.5 gene with the KanR cassette through homologous arms A and B. (3) Product after the first Red recombination. (4) The endonuclease I-SecI excised the Kana fragment, and the second Red recombination was completed through homologous arm A. (5) Illustration of the gene structure of DPV-BAC-δUL3.5. (**B**): Schematic diagram of the genome structure of DPV-BAC-RUL3.5. (1) Schematic of the gene structures of UL3 and UL4 in DPV-BAC-δUL3.5. A, B, and C are three homologous arms with sizes of 60 bp, 40 bp, and 40 bp, respectively. (2) The UL3.5 and KanR cassette gene sequences were amplified separately and fused into a single DNA fragment (UL3.5-KanR recombinant target fragment). In the first Red recombination, the UL3.5-KanR recombinant target fragment was inserted between the UL3 and UL4 genes through homologous arms A and B + C. (3) The product of the first Red recombination. (4) The Kana resistance gene was removed by the endonuclease I-SecI, and the second Red recombination was completed through homologous arm B. (5) Structural diagram of DPV-BAC-RUL3.5. (**C,D**): Rescue of the DPV-BAC-δUL3.5 deletion mutant and the DPV-BAC-RUL3.5 reverted recombinant virus strains. The correctly sequenced positive infectious clonal plasmids were transfected into DEFs to observe the formation of green viral fluorescent plaques. Scale bar: 50 μm. (**E**) PCR identification of DPV-BAC-δUL3.5 and DPV-BAC-RUL3.5. Viral DNA was extracted from cells infected with DPV-BAC, DPV-BAC-δUL3.5, and DPV-BAC-RUL3.5, and the UL3.5 gene was amplified using identification primers. Lane 1: Using DPV-BAC DNA as the template. Lane 2: Using DPV-BAC-δUL3.5 DNA as the template. Lane 3: Using DPV-BAC-RUL3.5 DNA as the template.

To investigate the role of DPV pUL3.5 in the viral life cycle, we constructed a recombinant BAC clone plasmid with a deletion of UL3.5 (DPV-BAC-δUL 3.5) and its revertant mutant (DPV-BAC-RUL3.5) (Figure 4A,B). Following sequence verification, these plasmids were transfected into DEFs for virus rescue. Green fluorescent spots emerged at 24 h post-transfection and gradually expanded within 72 h, consistent with the cell lesion (Figure 4C,D). Notably, the expansion rate of green fluorescence was slower in DPV-BAC-δUL3.5 compared to DPV-BAC-RUL3.5. Supernatants were collected and inoculated into fresh DEFs. After three blind passages, the supernatant and cell samples were collected. Viral DNA was extracted from the supernatant samples for PCR analysis. As shown in Figure 4E, the successful deletion of UL3.5 resulted in a 590 bp DNA band, matching the band in lane 2, while the DNA fragments amplified from DPV-BAC and DPV-BAC-RUL3.5 were 953 bp (lanes 1 and 3), indicating the presence of the intact UL3.5 gene (363 bp). Therefore, we successfully constructed and rescued the DPV-BAC-δUL3.5 and DPV-BAC-RUL3.5 recombinant viruses.

### 3.4. The Deletion of UL3.5 Leads to the Slow Growth Rate In Vitro

**Figure 5:**
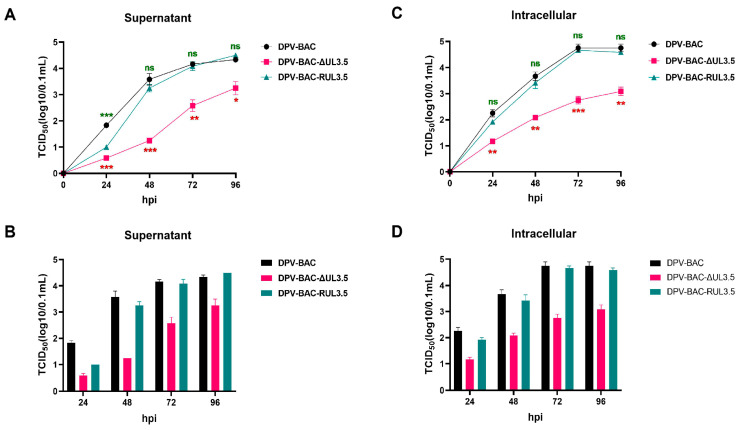
Growth kinetics in vitro. DEFs were infected with DPV-BAC, DPV-BAC-δUL3.5, or DPV-BAC-RUL3.5 viruses at 0.01 MOI. Supernatants and cell samples were collected at different time points for determination of virus titer (TCID_50_). Data dispersion was analyzed using mean ± SEM. (**A**,**B**): Virus titers of samples from the supernatant. (**C**,**D**): Virus titers of samples from the cells. Statistical differences in TCID_50_ among virus samples at various time points were analyzed using the *t*-test and represented as *p* > 0.05 (ns), *p* ≤ 0.05 (*), *p* ≤ 0.01 (**), *p* ≤ 0.001 (***).

To investigate the impact of UL3.5 on viral replication, the multi-step growth curve of DPV-BAC-δUL3.5 was analyzed. DEFs were infected with 0.01 MOI of DPV-BAC-δUL3.5, DPV-BAC, or DPV-BAC-RUL3.5. Supernatants and cell samples were collected at 24, 48, 72, and 96 hpi for viral titer (TCID_50_) determination. The viral multi-step growth curve was designed to recapitulate the complete replication cycle within host cells. At a low MOI, only a limited subset of cells is initially infected, while the majority remain uninfected, thereby enabling progeny virions to propagate through sequential rounds of infection in uninfected cells, generating a stepwise replication dynamic. The results showed that all three viruses exhibited similar growth patterns after infecting cells, with viral titers gradually increasing over time. However, as shown in Figure 5A–D, the viral titer of DPV-BAC-δUL3.5 was significantly lower than that of DPV-BAC starting from 24 hpi in both supernatants and cell samples. This finding indicates that the absence of UL3.5 impaired the ability of DPV to release mature virions into the supernatant and its growth in cells, which led to the slow growth rate of virus in vitro. This defect was restored in the revertant virus DPV-BAC-RUL3.5. Therefore, these results demonstrate that DPV pUL3.5 promotes viral replication.

### 3.5. UL3.5 Protein Participated in the Viral Secondary Envelopment and Virion Release Process

**Figure 6:**
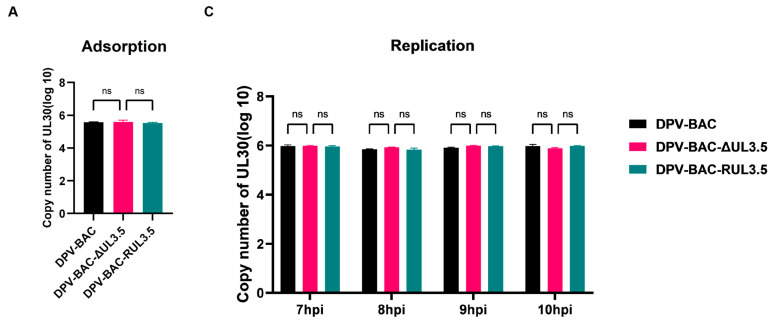

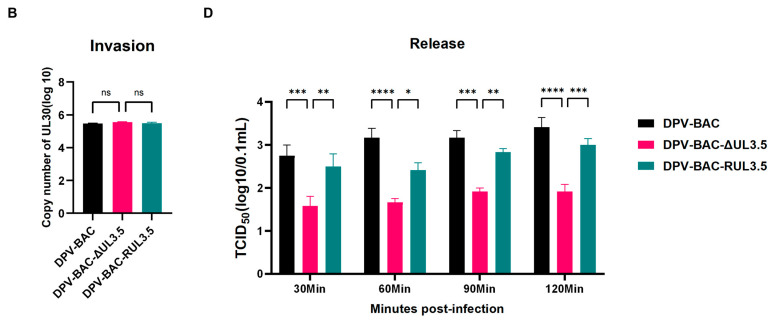
Effects of UL3.5 deletion on virus adsorption, invasion, replication, and release. (**A–C**): Cells were infected with 0.1 MOI of DPV-BAC, DPV-BAC-δUL3.5, or DPV-BAC-RUL3.5. Cellular samples were collected to extract viral DNA for determination of viral copy number, analyzing the effect of the UL3.5 gene on viral adsorption, invasion, and replication stages. (**D**): DEFs were infected with the viruses at 1 MOI. Cell supernatants were collected at specific time points to detect the virus titer (TCID_50_), analyzing the effect of the UL3.5 gene on the release stage of DPV. Statistical differences among samples were calculated using Analysis of Variance (ANOVA), represented as *p* > 0.05 (ns), *p* ≤ 0.05 (*), *p* ≤ 0.01 (**), *p* ≤ 0.001(***), and *p* ≤ 0.0001 (****).

Given the inhibitory effect of UL3.5 deletion on DPV’s in vitro growth kinetics, we further investigated the function of DPV pUL3.5 in the four phases of the viral life cycle: adsorption, invasion, replication, and release. We collected virus-infected cells, extracted virus DNA, and performed RT-qPCR to determine the copy number of the viral genome among DPV-BAC, DPV-BAC-RUL3.5, and DPV-BAC-δUL3.5. The results show that pUL3.5 did not affect the virus’s adsorption (Figure 6A) and invasion (Figure 6B), nor did it impact the virus’s replication process in cells (Figure 6C). Subsequently, we examined whether pUL3.5 affected the virus release process. As TCID_50_ assay detects cell-free infectious virions in supernatants and requires sufficient virion numbers to elicit statistically significant CPE, an MOI of 1 was employed to infect DEFs. Cells were infected with the virus for 18 h, followed by replacement with maintenance medium containing 2% FBS. Then supernatants were harvested at 30, 60, 90, and 120 min post-medium replacement for TCID_50_ assays. Results, as shown in Figure 6D, indicate that, with increasing time, more virions were released into the supernatant, and the viral titers of all three strains increased accordingly. However, the viral titer of DPV-BAC-δUL3.5 was continuously and substantially lower than that of DPV-BAC and DPV-BAC-RUL3.5, suggesting that pUL3.5 is essential for virion release from cells into the extracellular environment.

**Figure 7:**
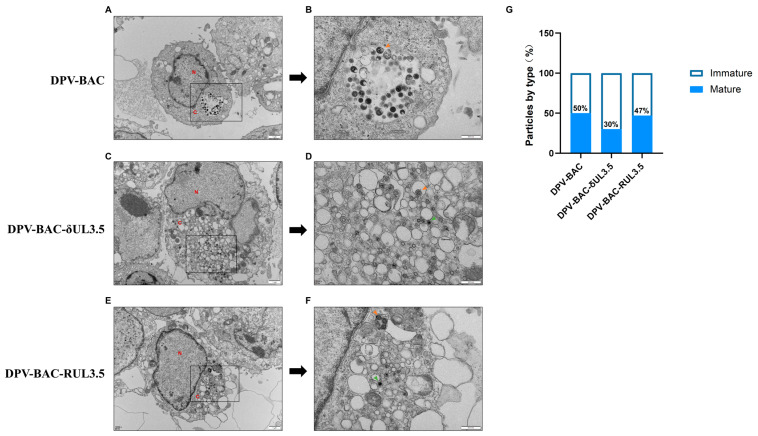
Electron microscopy analysis of DPV-BAC, DPV-BAC-δUL3.5, and DPV-BAC-RUL3.5 (original figures see Supplementary Materials). (**A**): The electron microscopic pictures of DEFs infected with DPV-BAC. Scale bar: 1 μm. (**B**): Higher magnifications with close-ups of selected areas. Yellow arrowheads indicate completely enveloped particles for DPV-BAC. Scale bar: 500 nm. (**C**): The electron microscopic pictures of DEFs infected with DPV-BAC-δUL3.5. (**D**): Higher magnifications with close-ups of selected areas. Green arrowheads indicate incompletely enveloped particles for DPV-BAC-δUL3.5. (**E**): The electron microscopic pictures of DEFs infected with DPV-BAC-RUL3.5. (**F**): Higher magnifications with close-ups of selected areas. Yellow arrowheads indicate completely enveloped particles for DPV-BAC-RUL3.5. (**G**): Ultrastructural quantification of secondary envelopment stages of DPV particles in infected DEFs by virus.

Following genome replication in the nucleus, herpesvirus DNA is packaged into preformed capsids to assemble nucleocapsids. These nascent nucleocapsids subsequently pass through the nuclear membrane and enter the cytoplasm. During this process, primary envelopment and de-envelopment are completed. Maturation concludes with secondary envelopment, where tegument-coated capsids acquire their final envelope by budding into trans-Golgi network (TGN)-derived and endosomal vesicles, yielding infectious mature virions [41]. To investigate the role of DPV pUL3.5 in viral morphogenesis, transmission electron microscopy (TEM) was performed on infected cells. As shown in Figure 7, DPV-BAC and DPV-BAC-RUL3.5 demonstrated normal secondary envelopment within the cytoplasm, generating mature virions (indicated by yellow arrowheads in Figure 7B,F). In contrast, UL3.5-deficient mutants exhibited cytoplasmic accumulation of non-enveloped nucleocapsids (green arrowheads in Figure 7D), indicative of aborted secondary envelopment. Quantitative analysis of cytoplasmic mature and immature viral particles is presented in Figure 7G. The percentages of mature virions in DPV-BAC- and DPV-BAC-RUL3.5-infected cells were 50% and 47%, respectively. In contrast, only 30% of particles reached maturity in DPV-BAC-δUL3.5-infected cells. These findings demonstrate that pUL35 facilitates secondary envelopment during virion morphogenesis, with its absence significantly compromising maturation efficiency.

### 3.6. UL3.5 Exerts a Significant Impact on Cell-to-Cell Spread

**Figure 8:**
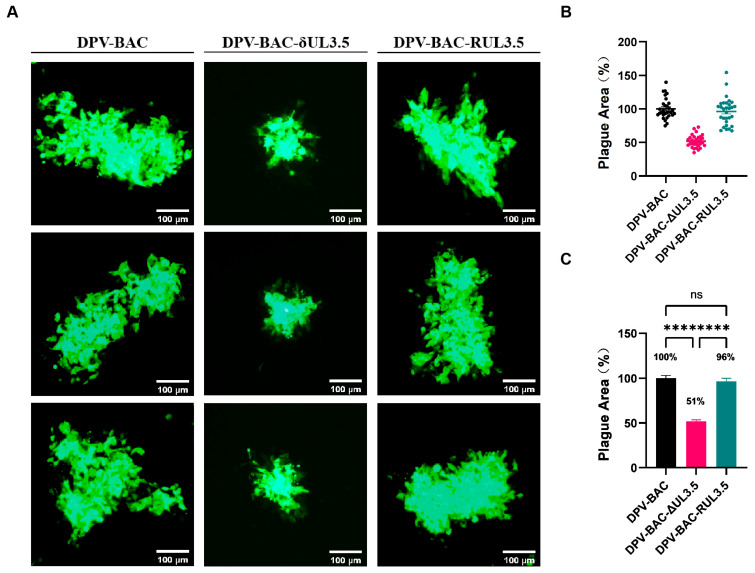
Impact of UL3.5 on DPV cell-to-cell spread. DEFs were infected with 0.005 MOI of DPV-BAC, DPV-BAC-δUL3.5, or DPV-BAC-RUL3.5 for 2 h (original figures see Supplementary Materials). The cells were then overlaid with a mixture of 2× DMEM containing 2% serum and 2% agarose to restrict viral spread. Fluorescent plaques were observed under a fluorescence microscope. (**A**): Viral fluorescent plaques produced after infection of cells with the three virus strains. Scale bar: 100 μm. (**B,C**): Thirty plaques were randomly selected for statistical analysis of their areas, and the average size of DPV-BAC plaques set to 100%. One-way ANOVA was used to analyze statistical differences among the groups, with significance levels indicated as *p* > 0.05 (ns) and *p* ≤ 0.0001 (****).

To investigate the role of pUL3.5 in DPV cell-to-cell transmission, DEFs were pre-cooled at 4 °C for 1 h, inoculated with 0.005 MOI of viruses at 4 °C for 2 h to synchronize infection, washed three times with 4 °C pre-cooled PBS, and overlaid with a mixture of 2× DMEM containing 2% serum and 2% agarose to restrict viral spread. After 72 h incubation at 37 °C, fluorescent plaques generated by the viruses were observed. We found that the fluorescent plaques of DPV-BAC-δUL3.5 were significantly smaller than those of DPV-BAC and DPV-BAC-RUL3.5 (Figure 8A). A total of 30 fluorescent plaques generated by each virus were randomly selected, and the plaque area was statistically analyzed. As shown in Figure 8B,C, the average plaque area of DPV-BAC was considered 100%. In contrast, the average plaque size produced by DPV-BAC-δUL3.5 was 51% of the wild-type size, representing a 49% reduction compared to DPV-BAC. The average green fluorescent plaque area of DPV-BAC-RUL3.5 was 96%, which did not differ significantly from the average plaque area of DPV-BAC. A low MOI was intentionally selected in this experimental design to attenuate progression into the exponential growth phase of viral replication. This strategy effectively prevented plaque coalescence caused by rapid viral propagation, thereby maintaining discrete plaque morphology essential for accurate enumeration and quantitative analysis. These data indicate that pUL3.5 is crucial for the cell-to-cell spread of DPV.

## 4. Discussion

The genome of DPV is arranged in the order of UL-IRS-US-TRS, which is a typical genome arrangement of D-type herpesvirus. Herpesvirus genes adhere to a strict temporal expression pattern governed by cascade regulation. Based on their transcriptional timing, these genes are classified into immediate early gene (IE or α), early gene (E or β) and late gene (L or γ), encoding immediate early protein, early protein, and late protein, respectively. Differential temporal transcription orders imply distinct functional roles for these genes [42]. While certain genes of DPV have been relatively well-characterized, the functional classification and roles of other genes and their encoded proteins remain unconfirmed. Herein, we investigated the DPV UL3.5 gene and its encoded protein (pUL3.5). By analyzing UL3.5 transcriptional and protein levels at sequential time points post-DPV infection, we found that the expression kinetics of UL3.5 were similar to those of the early protein UL28. A drug inhibition assay of GCV and CHX further showed that DPV UL3.5 was an early viral gene.

**Figure A1:**
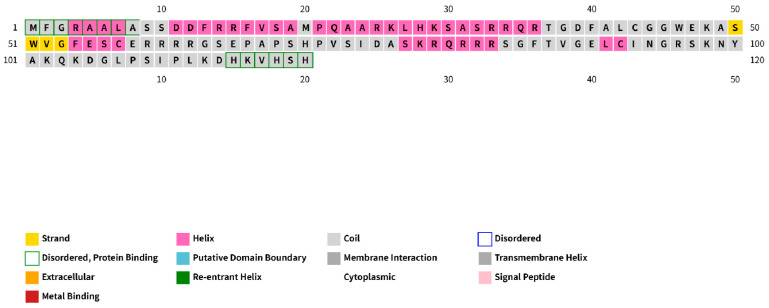
Prediction of intrinsically disordered regions (IDRs) in DPV pUL3.5. The UL3.5 protein, consisting of 120 amino acids, was analyzed for potential IDRs. Green boxes highlight amino acids within the IDRs predicted to mediate protein binding. These residues are mainly concentrated in the terminal 10-amino-acid regions flanking both ends of the polypeptide chain.

We also found that DPV pUL3.5 is mainly localized in the cytoplasm with a punctate distribution pattern and is closely related to various subcellular organelles, including the ER and mitochondria. It is particularly intriguing that DPV pUL3.5 exhibits a punctate distribution within the cytoplasm, prompting us to consider the phenomenon of liquid–liquid phase separation (LLPS). Recent studies have suggested that LLPS may serve as the physicochemical basis for the formation of membraneless organelles in cells, such as *p* granules, nucleoli, and stress granules [43]. LLPS has also been reported to play a crucial role in the pathogenesis and progression of various diseases, including cancer, metabolic disorders, and neurodegenerative diseases [44]. The occurrence of liquid–liquid phase separation may be analogous to amyloid formation, as only a small subset of proteins can undergo amyloid transformation under physiological conditions and, similarly, only specific proteins may possess the ability to form phase-separated structures within living cells [45]. The academic community has proposed the “scaffolds and clients” theory through investigations into the common characteristics of molecules capable of undergoing phase separation under physiological conditions [46]. Scaffold molecules are considered the driving force behind phase separation, whereas the proteins recruited into the liquid droplets after phase separation are termed client proteins. This process relies on an interaction network formed by protein–protein interactions and protein–RNA interactions. Two classes of proteins are involved in promoting the formation of such interaction networks: one characterized by multiple folded domains [47] and the other featuring intrinsically disordered regions (IDRs) [48,49,50,51]. An IDR, also known as a low complexity domain (LCD), is typically enriched with specific amino acid residues such as polar, aromatic, and/or charged amino acids. It exhibits high conformational flexibility and facilitates the formation of multivalent weak intermolecular/intramolecular interactions, serving as a critical factor driving protein phase separation [52]. IDR prediction analysis of DPV pUL3.5 revealed potential intrinsically disordered regions (IDRs) capable of protein binding (Figure A1), and the presence of IDRs may be implicated in the initiation and regulation of phase separation. Furthermore, the punctate cytoplasmic distribution of pUL3.5 resembles phase-separated condensates observed in other herpesviruses (e.g., HSV-1 ICP4 [53]). Although this pattern alone cannot conclusively confirm LLPS, it suggests that pUL3.5 may participate in organizing viral replication compartments through dynamic aggregation. These findings suggest that pUL3.5 may contribute to viral assembly via dynamic subcellular clustering, a process that could involve phase separation or other cooperative binding mechanisms. Given that the genetic and biological properties of phase separation remain incompletely characterized, caution is warranted when speculating about the occurrence of LLPS. Future studies will employ in vitro phase separation assays (e.g., PEG-induced condensation) and fluorescence recovery after photobleaching (FRAP) to directly test whether pUL3.5 undergoes LLPS under physiological conditions.

The life cycle of herpesviruses can be broadly divided into four stages: adsorption, invasion, replication, and release. Additionally, secondary envelopment occurring in the cytoplasm is critical for the maturation of viral particles. Nucleocapsids in the cytoplasm bud into specialized vesicles enriched with viral glycoproteins to form mature virions. The vesicles required for herpesvirus secondary envelopment are primarily derived from the trans-Golgi network (TGN) and early endosomes. These vesicles harbor most viral glycoproteins and a subset of membrane-associated tegument proteins. Interactions between tegument proteins bind to the nucleocapsid and those on the vesicle membrane facilitate membrane fusion, thereby driving the completion of secondary envelopment [15]. Virus-encoded proteins play critical roles in distinct stages of the viral life cycle. For example, UL49.5 affects DPV adsorption, as well as subsequent invasion and spread [54], while VP22 regulates virion assembly and the cell-to-cell spread of DPV [55]. By deleting the UL3.5 gene, we explored its impact on DPV growth and the replication cycle. The results show that the DPV UL3.5 gene is not essential for virus production, but its deletion affects the growth of virus in vitro and inhibits the virus release process. The UL3.5 gene is conserved in a genomic location downstream of UL3, but exhibits high variability in size and sequence among different members of the *Alphaherpesvirinae* subfamily, and is absent from the herpes simplex virus genome [28]. The predicted molecular size of UL3.5 protein ranges from 71 amino acids (aa) in VZV to 224 aa in PrV, with low overall amino acid sequence homology. Notably, all predicted UL3.5 gene products share a short segment of highly conserved amino acids near the N-terminal, potentially indicating a conserved functional roles [25]. In PrV, the UL3.5 gene encodes a non-structural protein required for virus particles to acquire a secondary envelope in the cytoplasm. Deletion of PrV pUL3.5 leads to accumulation of naked nucleocapsids in the cytoplasm, significantly reducing the release of infectious virions [27]. Given that pUL3.5 modulates DPV release, we aimed to investigate its potential role in viral assembly. Electron microscopy analysis of viral particle morphology revealed that cells infected with DPV-BAC-δUL3.5 exhibited accumulations of unenveloped nucleocapsids clustered around intracellular vesicles, indicating that UL3.5 deletion disrupts secondary envelopment. This defect likely impedes the trafficking of mature virions from intracellular compartments to the extracellular space, thereby suppressing viral egress.

Viruses spread to adjacent uninfected cells via two primary mechanisms: cell-free transmission and cell-to-cell transmission [56]. Herpesviruses primarily utilize cell-to-cell transmission, a strategy that circumvents host immune detection and elimination by avoiding extracellular virion release, thereby improving the efficiency of viral infection and transmission within the host [57]. Previous studies have shown that DPV UL54, US3, and VP22 proteins facilitate viral cell-to-cell spread [55]. In this study, the DPV-BAC-δUL3.5 deletion mutant produced significantly smaller plaques than the wild-type (DPV-BAC) and the revertant (DPV-BAC-RUL3.5). For example, wild-type HSV-1 forms large plaques in cell cultures, whereas gE- and gI-mutants—defective in cell-to-cell spread—exhibit a significant reduce plaque area due to replication-associated diffusion defects [58]. Similarly, PrV UL3.5 deletion inhibits virion release and reduces plaque formation in cell cultures [12]. Notably, VZV—a stringent cell-to-cell transmitted virus—shows no significant impact on replication or spread following deletion of its homologous ORF57 gene [20]. Our results indicate that DPV pUL3.5 affects viral cell-to-cell spread. We infer that UL3.5 may act mainly on virus release from the cell surface, but further investigation into the mechanisms through which DPV pUL3.5 promotes viral cell-to-cell spread is needed.

This study has several limitations that warrant consideration. Although DPV pUL3.5 was shown to regulate virion assembly, the exact molecular partners (e.g., viral tegument proteins or host trafficking machinery) mediating this process remain to be mapped via proteomic approaches.

## 5. Conclusions

**Figure 9:**
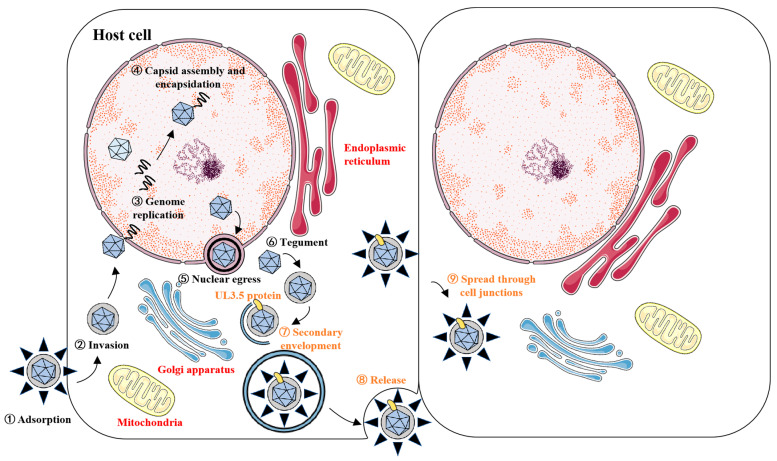
Academic schematic model depicting the critical role of UL3.5 in the DPV life cycle: secondary envelopment, release, and cell-to-cell spread.

In this study, we demonstrated that the DPV UL3.5 gene-encoded protein (pUL3.5) exhibits a cytoplasmic punctate distribution and is co-located with mitochondria and the ER. In addition, we found that DPV UL3.5 is an early gene encoding an early viral protein, plays a critical role in the DPV life cycle by regulating the viral secondary envelopment and release, and has an important effect on the cell-to-cell spread of DPV (Figure 9).

## Data Availability

All data analyzed during this study are available from the corresponding author upon reasonable request.

## Supplementary Materials

The following supporting information can be downloaded at: https://www.mdpi.com/article/10.3390/vetsci12060510/s1.

## Abbreviations

The following abbreviations are used in this manuscript:

aa	Amino acid
AnHV-1	Anas herpesviruses 1
BAC	Bacterial artificial chromosome
BHV-1	Bovine herpesvirus 1
cDNA	Complementary DNA
CHX	Cycloheximide
DEFs	Duck embryo fibroblast
DEV	Duck enteritis virus
DMEM	Dulbecco Modified Eagle Medium
DNA	Deoxyribonucleotide acid
DP	Duck plague
DPV	Duck plague virus
ER	Endoplasmic reticulum
EGFP	Enhanced green fluorescent protein
FBS	Fetal bovine serum
GCV	Ganciclovir
HSV-1/2	Herpes simplex virus 1/2
ILTV	Infectious laryngotracheitis virus
MOI	Multiplicity of infection
NanoLuc	NanoLuc^®^ luciferase
ORF	Open reading frame
PBS	Phosphate-buffered saline solution
PBST	Phosphate-buffered saline with Tween
PCR	Polymerase chain reaction
RNA	Ribonucleic Acid
RT-qPCR	Quantitative real-time PCR
TBST	Tris-buffered saline with Tween

## Appendix A

**Table A1:** The sequence of primers.

Plasmid/Gene	Primer	Primer Sequence (5′ → 3′)
pEGFP-N1-UL3.5	F	AGATCTCGAGCTCAAGCTTCGAATTCGCCACCATGTTCCGGGCGCGGCATT
	R	GACCGGTGGATCCCGGGCCCGCGGTACCGTAATGAGAATGAACCTTATGAT
DPV-BAC-NanoLuc-UL3.5	F	TTTACATCGGCGACAACGTAATAGAGGTCAGTACGCCTCAGTCCTGAAGTGATCGCGAACTATGGATTACAAGGACGACGATGACAAGAACTCCTTCTCC
	R	GCGATTGCTGGTCTATGAGTCGATAACGGCCGGTGTTGATGATTACCTGGTTCAGGCCGCTCGTTTCCGCCGAGTGATCCAGCCAGTGTTACAACCAAT
DPV-BAC-δUL3.5	F	TAGAGGTCAGTACGCCTCAGTCCTGAAGTGATCGCGAACTTAGGGATAACAGGGTAATCGATTT
	R	ATTACCTGGTTCAGGCCGCTCGTTTCCGCCGAGTGATCCAAGTTCGCGATCACTTCAGGACTGAGGCGTACTGACCTCTAGCCAGTGTTACAACCAAT
DPV-BAC-RUL3.5	F	TTACATCGCCGACAACGTAATAGAGGTCAGTACGCCTCAGTCCTGAAGTGATCGCGAACTATGTTCCGGGCGCGGCATTA
	R	GCGATTGCTGGTCTATGAGTCGATAACGGCCGGTGTTGATGATTACCTGGTTCAGGCCGCTCGTTTCCGCCGAGTGATCCAGCCAGTGTTACAACCAAT
Identification of UL3.5	F	GTGGCCACGGCAATAAAAGGC
	R	GTGAGTCGTCATCTAGAACGG
ICP27 gene [55]	F	TTGCTCGACCTAAGACAGGA
	R	TCATTAGCAGTAGTTCCGCG
UL28 gene [55]	F	TTAAGCTAAGCCGTGCCGAT
	R	TGGAGCCAGTTGTTGAAGCA
UL47 gene [55]	F	AACGGAGTTGCTTGGAGAACA
	R	TGGGCGATGAAACAGAGTAGG
β-actin [40]	F	GATCACAGCCCTGGCACC
	R	CGGATTCATCATACTCCTGCTT
UL30 gene [55]	F	TTTCCTCCTCCTCGCTGAGTG
	R	GTGAGTCGTCATCTAGAACG

**Table A2:** Cell viability under GCV/CHX concentrations.

Drug Treatment	Concentration	OD450 Values (mean ± SD)	Cell Viability
DMSO	0.2%	2.91 ± 0.08	100.00%
GCV	300 μg/mL	2.89 ± 0.01	99.33%
CHX	200 μg/mL	2.89 ± 0.06	99.64%
GCV + CHX	300 μg/mL + 200 μg/mL	2.88 ± 0.01	99.15%
