# Supplementary material for: The Protein Encoded by the UL3.5 Gene of the Duck Plague Virus Affects Viral Secondary Envelopment, Release, and Cell-to-Cell Spread
Source: Vet Sci. 2025 May 23;12(6):510. doi: 10.3390/vetsci12060510 (PMC12197417; doi:10.3390/vetsci12060510)
Supplement: Supplementary file 1 [file vetsci-12-00510-s001.zip › vetsci-3546009-supplementary.pdf]

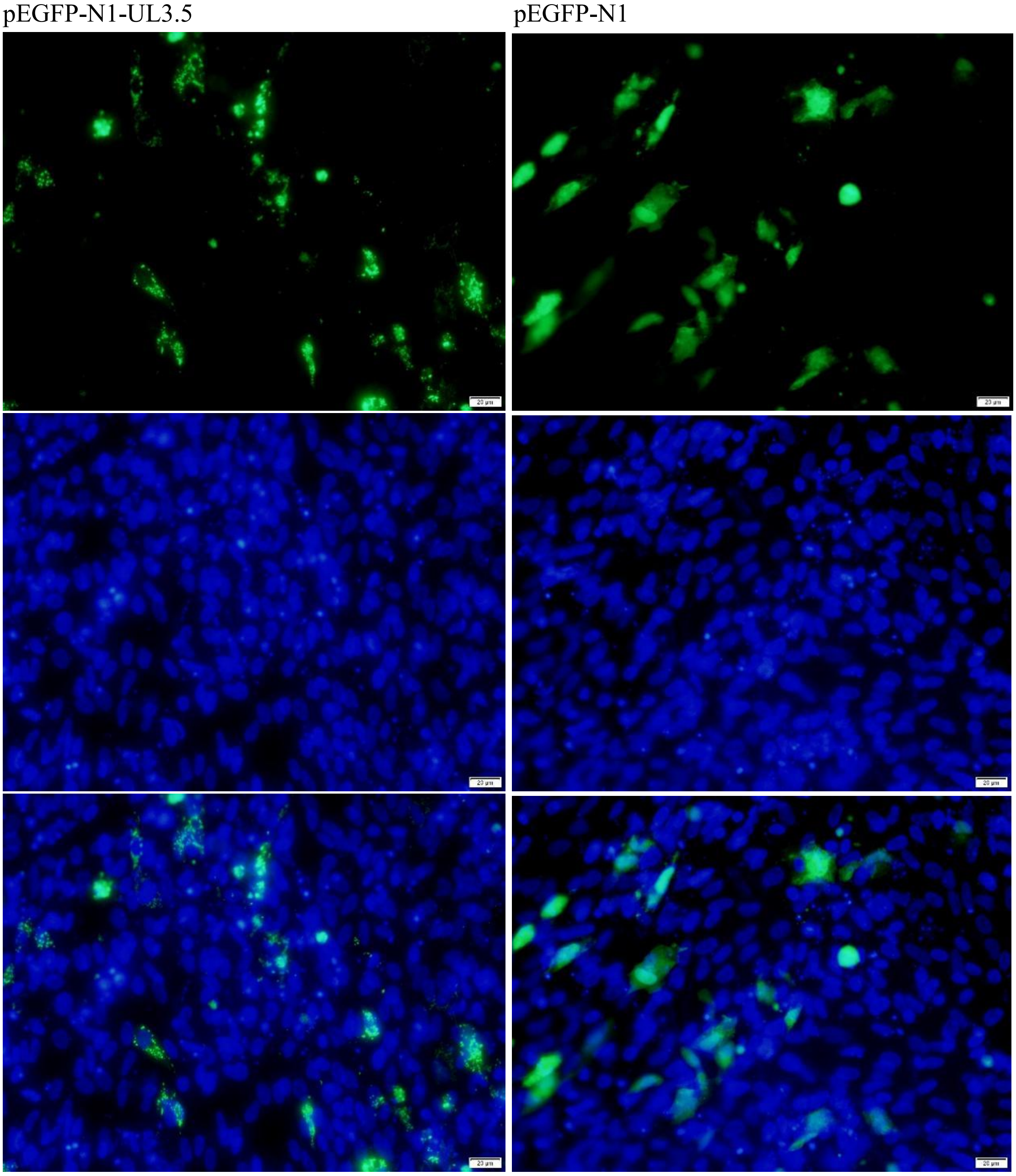

Figure 1. (**A**): DPV pUL3.5 is distributed in cytoplasm.

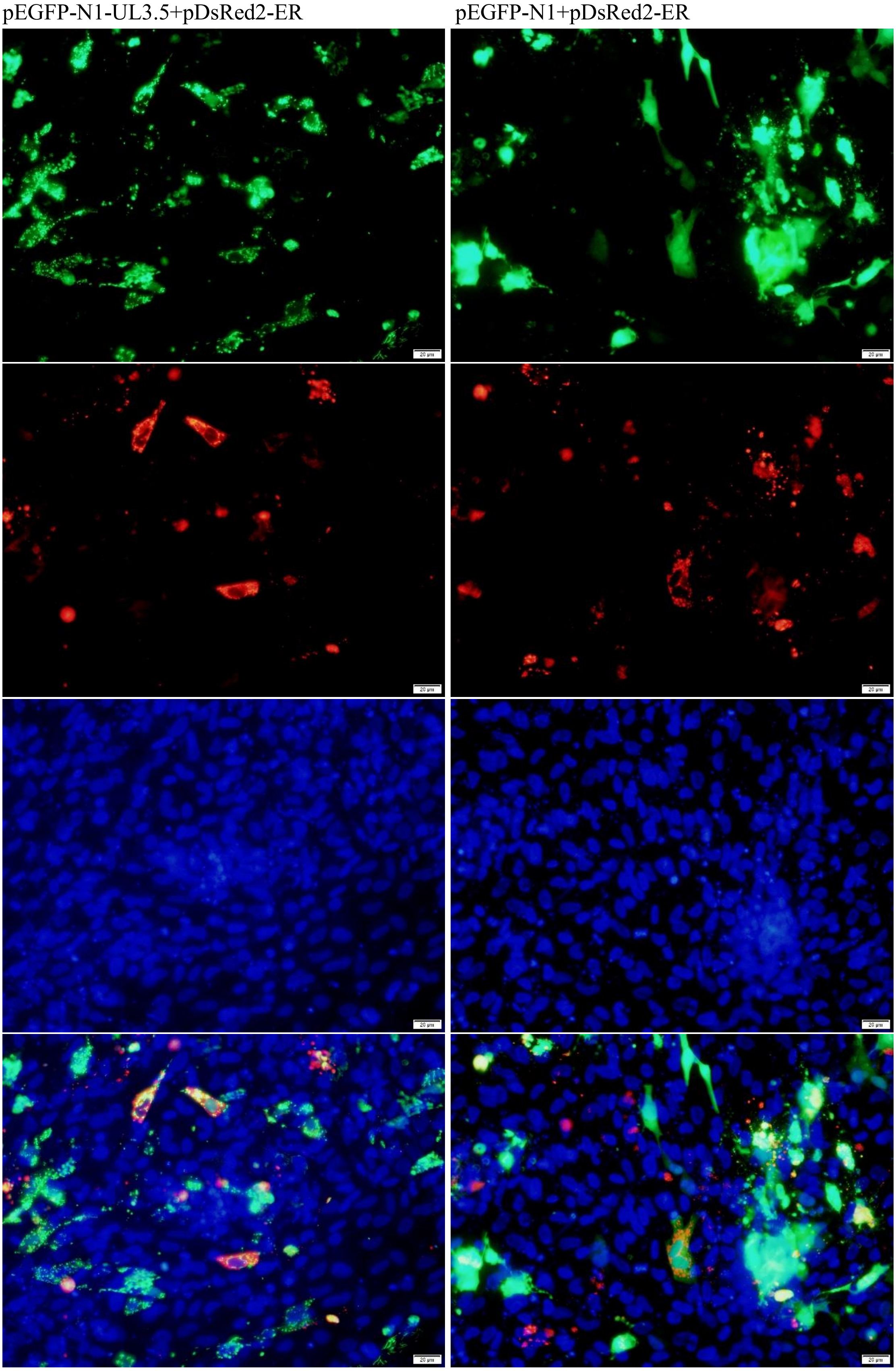

Figure 1. (**B**): DPV pUL3.5 co-localizes with endoplasmic reticulum.

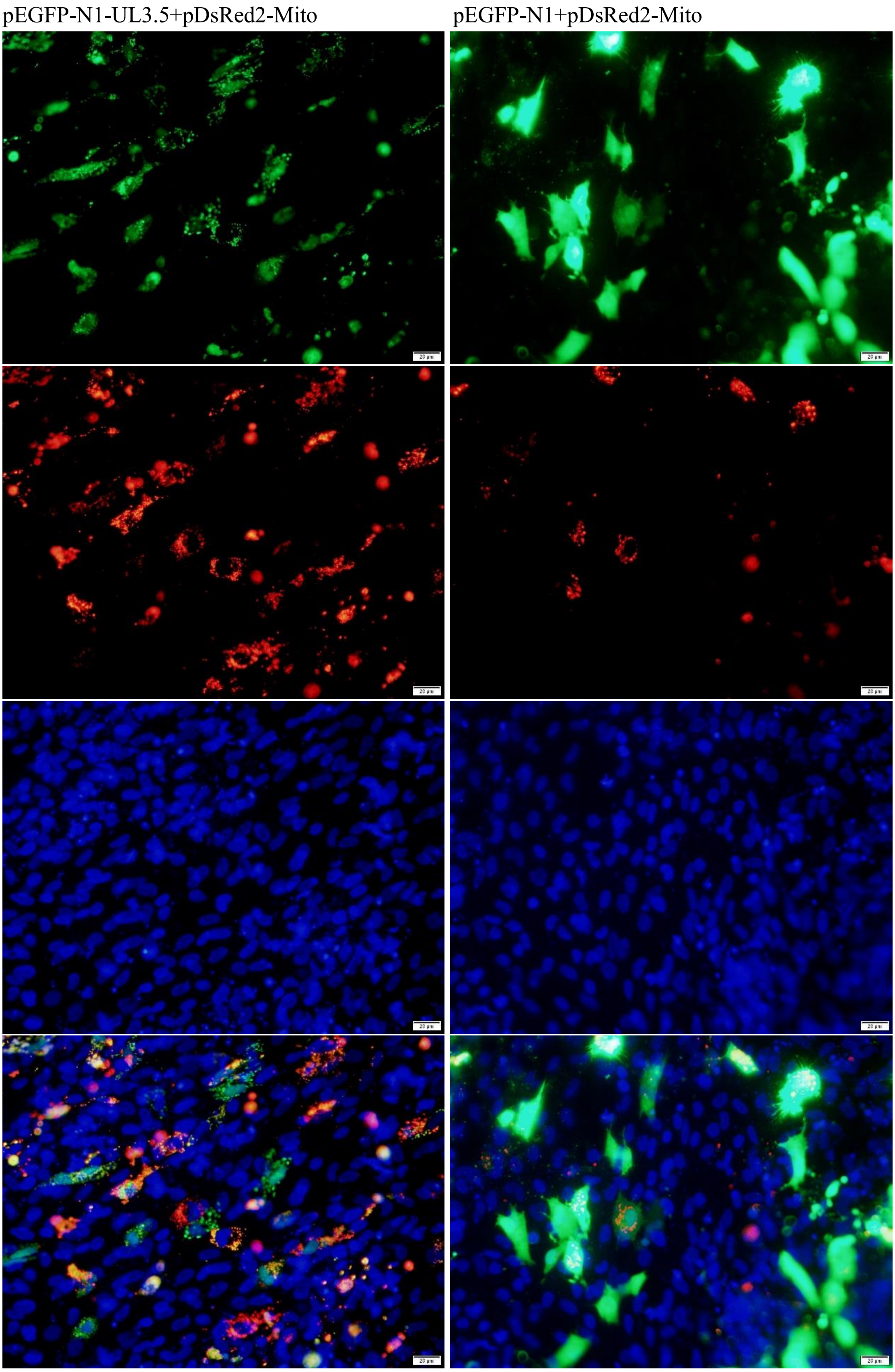

Figure 1. (**D**): DPV pUL3.5 co-localizes with mitochondria.

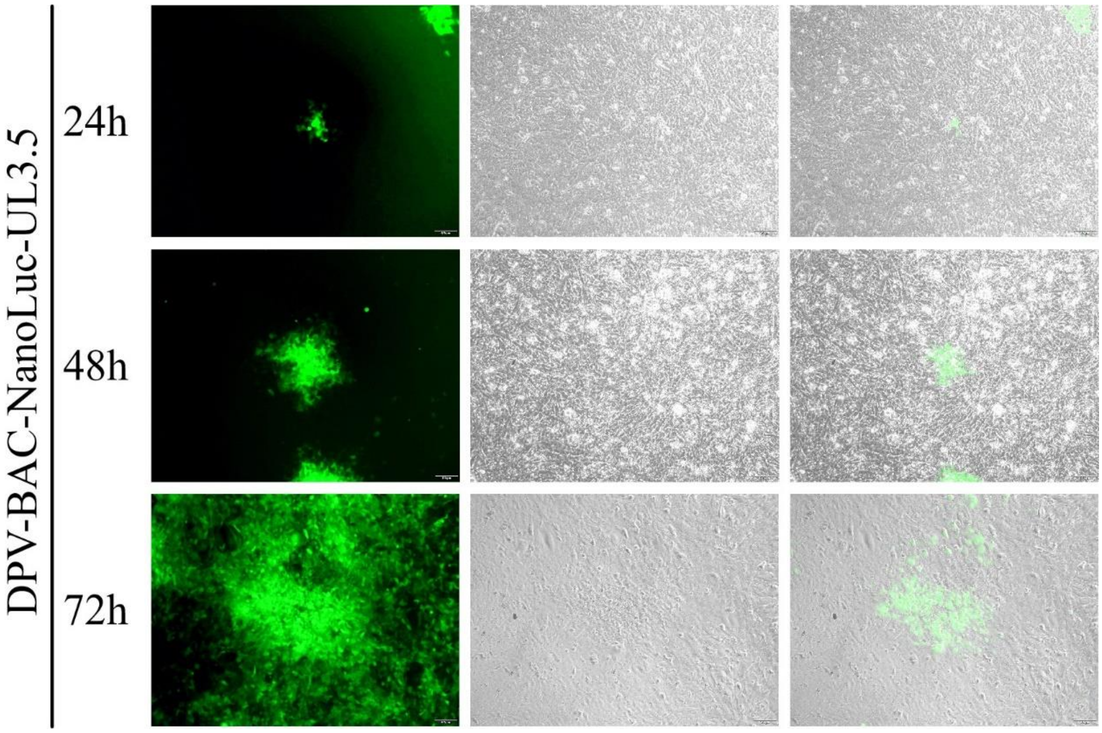

Figure 2. (**B**): Rescue of the DPV-BAC-NanoLuc-UL3.5 recombinant strain.

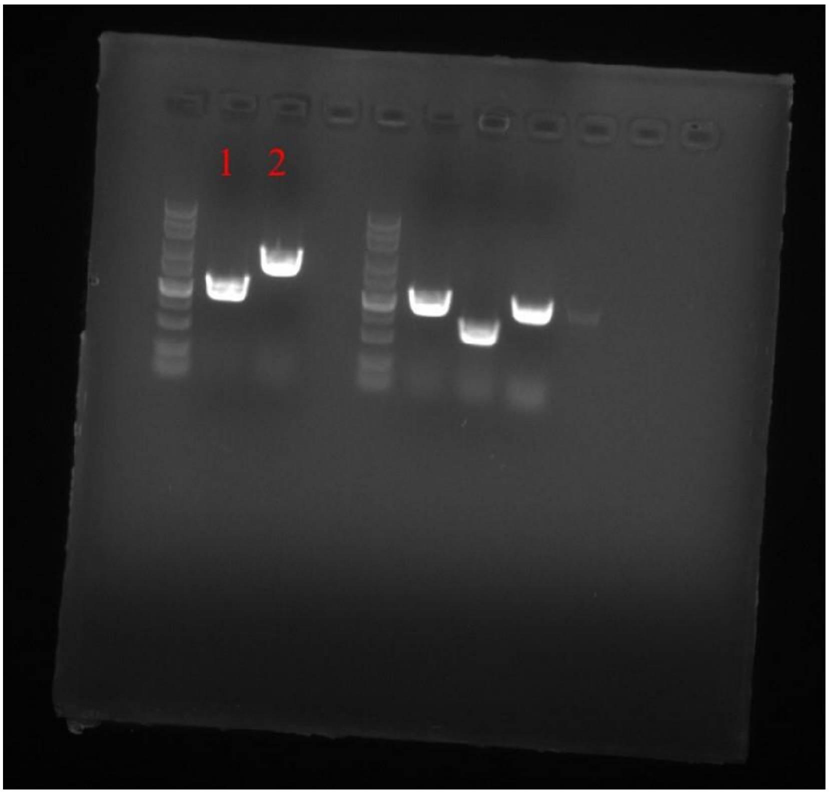

Figure 2. (**C**): PCR identification of DPV-BAC-NanoLuc-UL3.5. Lane 1: Using DPV-BAC DNA as the template; Lane 2: Using DPV-BAC-NanoLuc-UL3.5 DNA as the template.

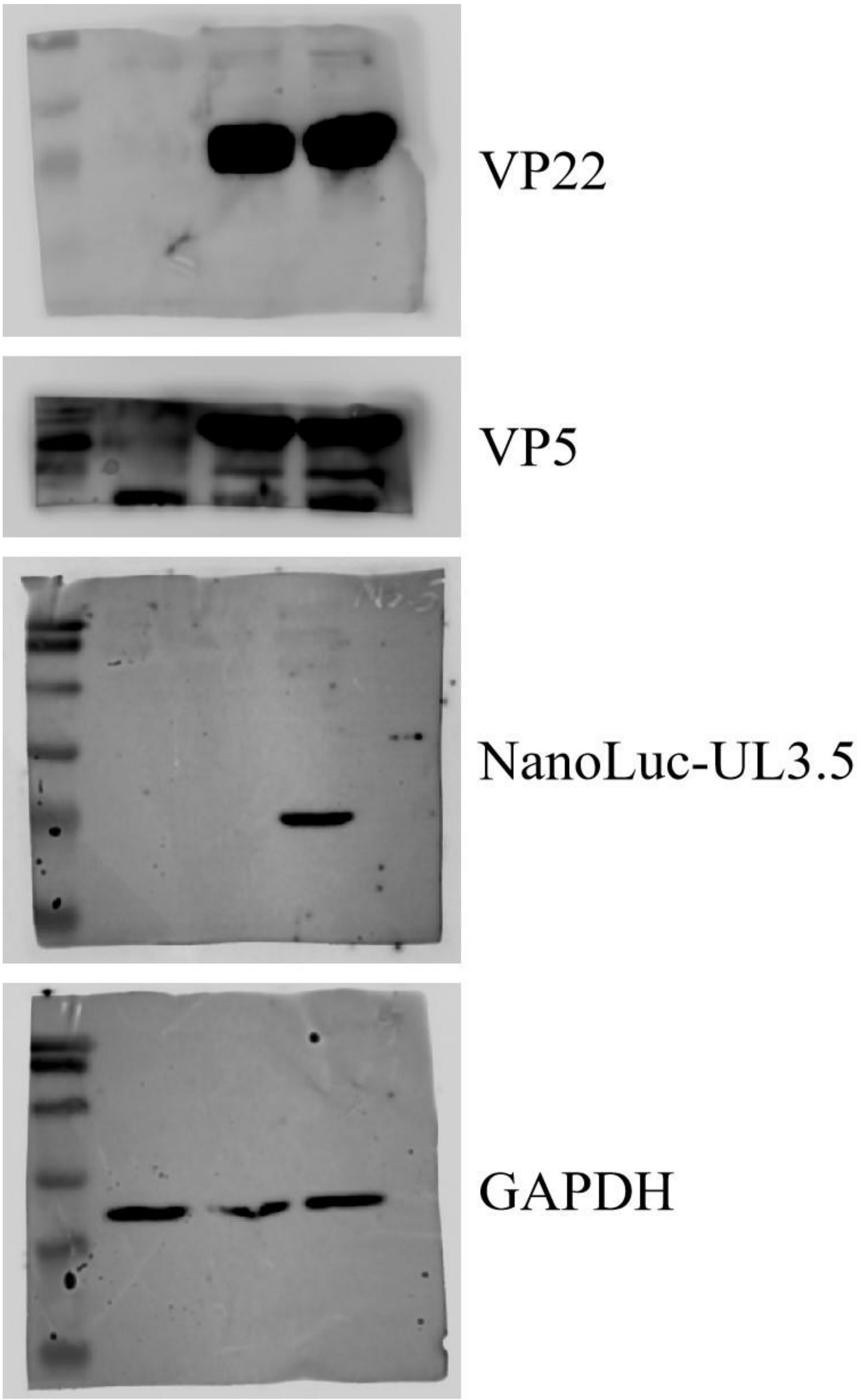

Figure 2. (**D**): WB identification of DPV-BAC-NanoLuc-UL3.5. VP22 and VP5 were used as infection controls, and GAPDH served as internal reference control for the experiment.

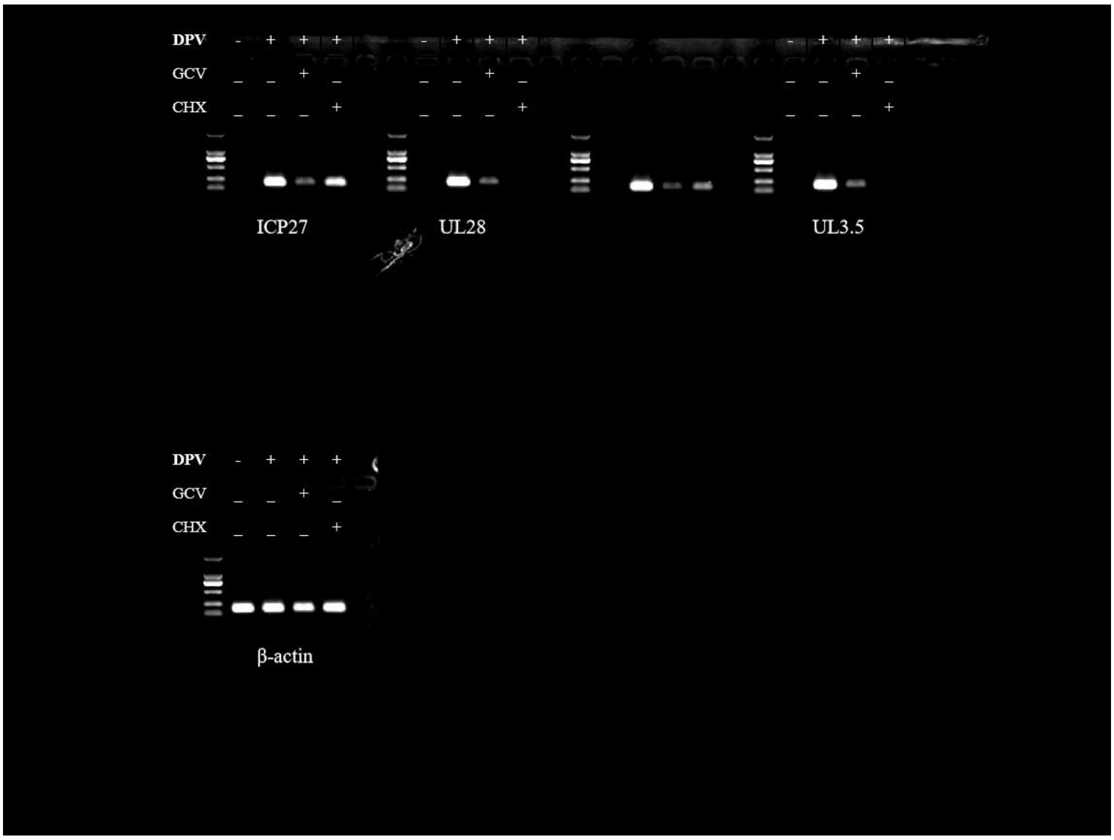

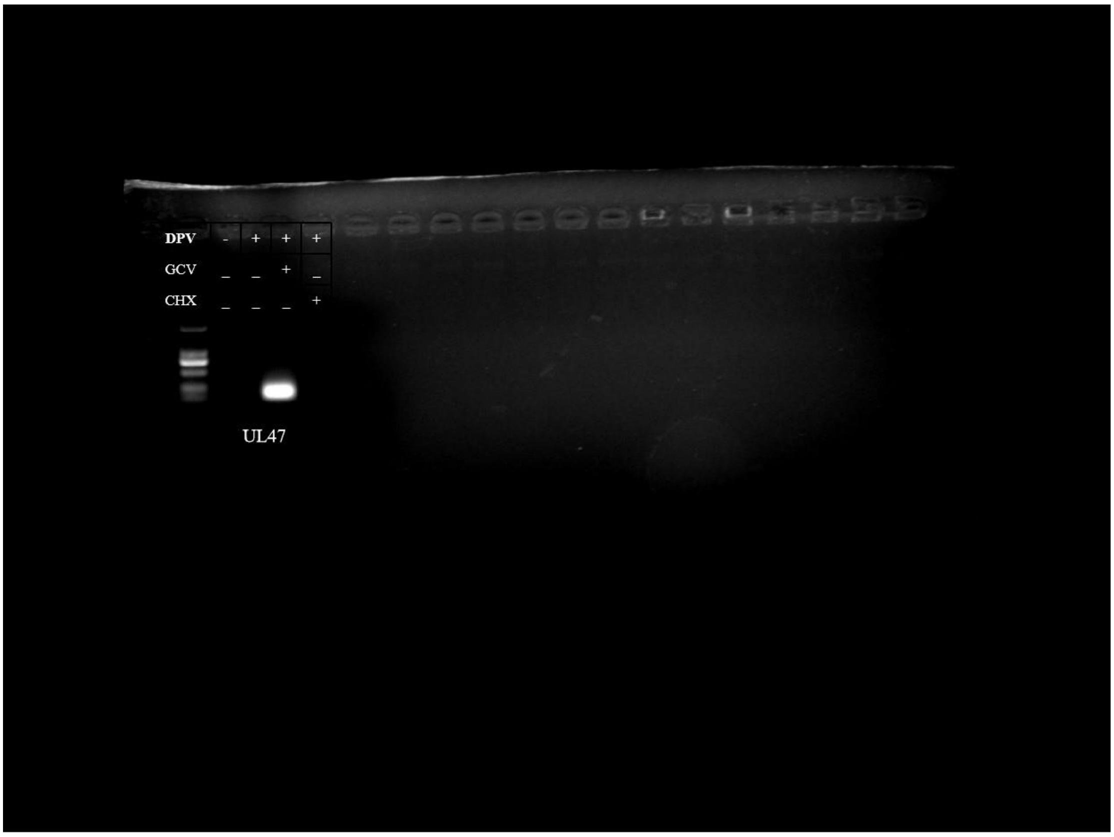

Figure 3. (**E**): Detection of the UL3.5 genotype after GCV/CHX treatment. ICP27, UL28, and UL47 respectively represent IE, E, and L genes.

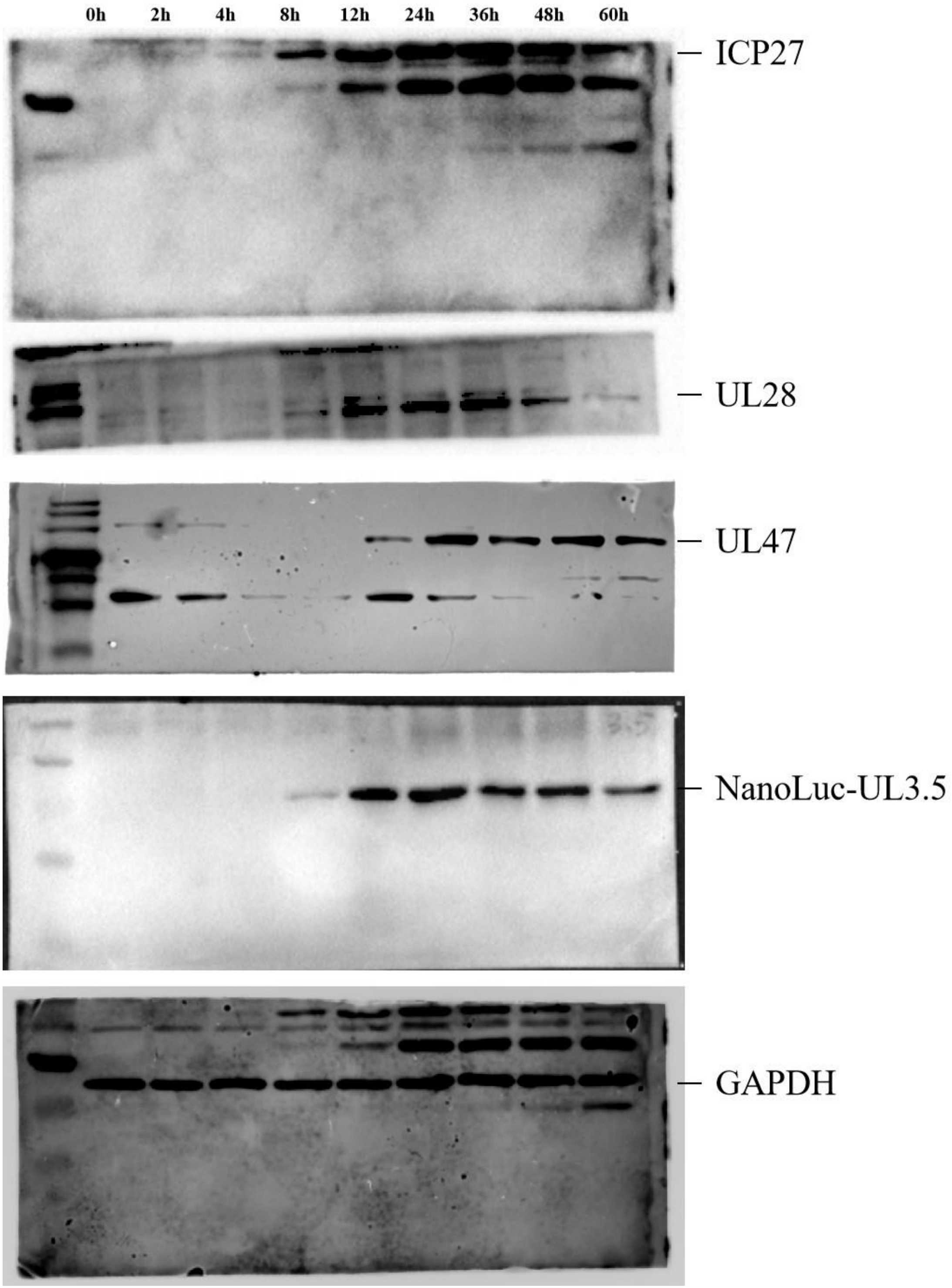

Figure 3. (**F**): Western Blotting was used to detect the expression timing of each protein. ICP27, UL28, and UL47 respectively represent IE, E, and L proteins, while GAPDH served as the internal reference protein.

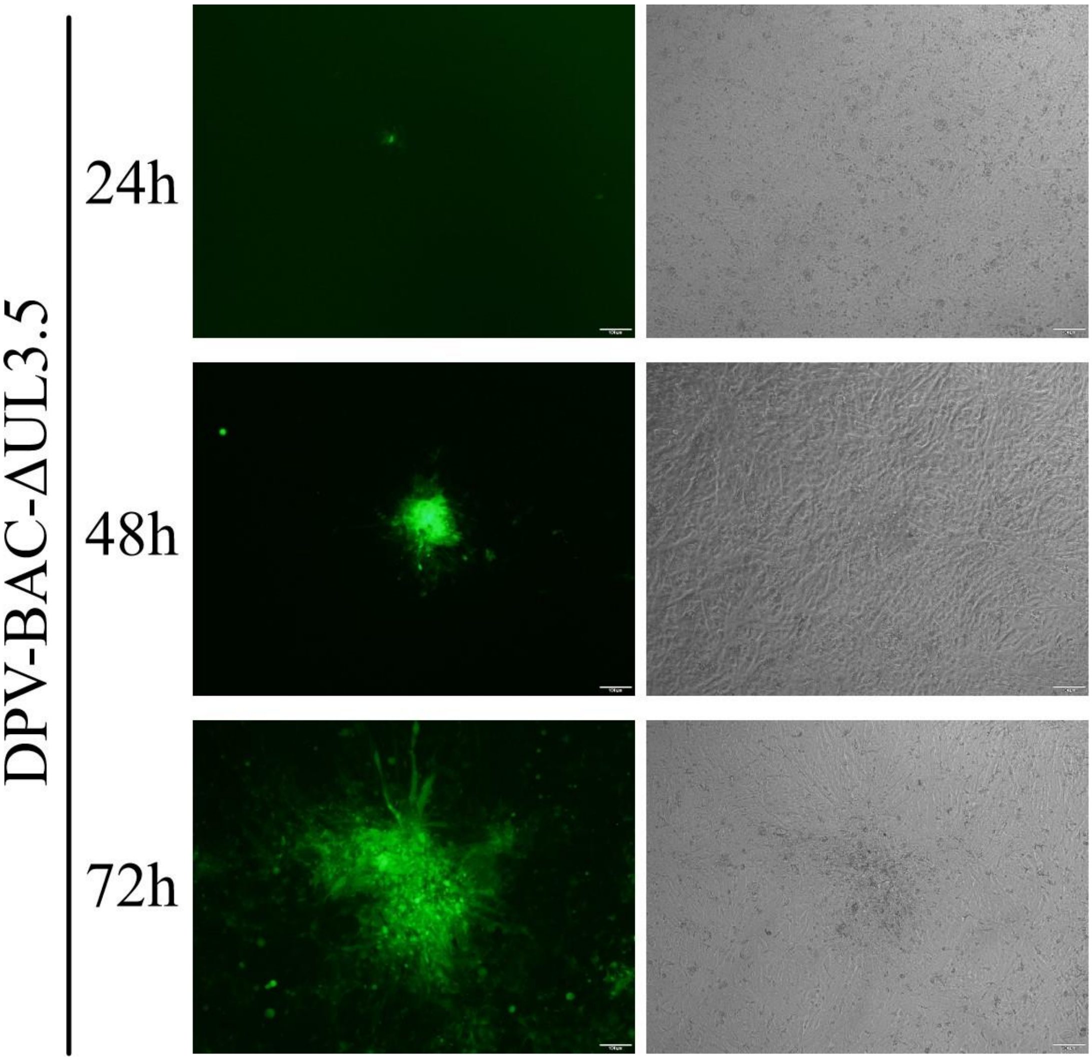

Figure 4. (**C**): Rescue of the DPV-BAC-δUL3.5 deletion mutant. The correctly sequenced positive infectious clonal plasmids were transfected into DEFs to observe the formation of green viral fluorescent plaques.

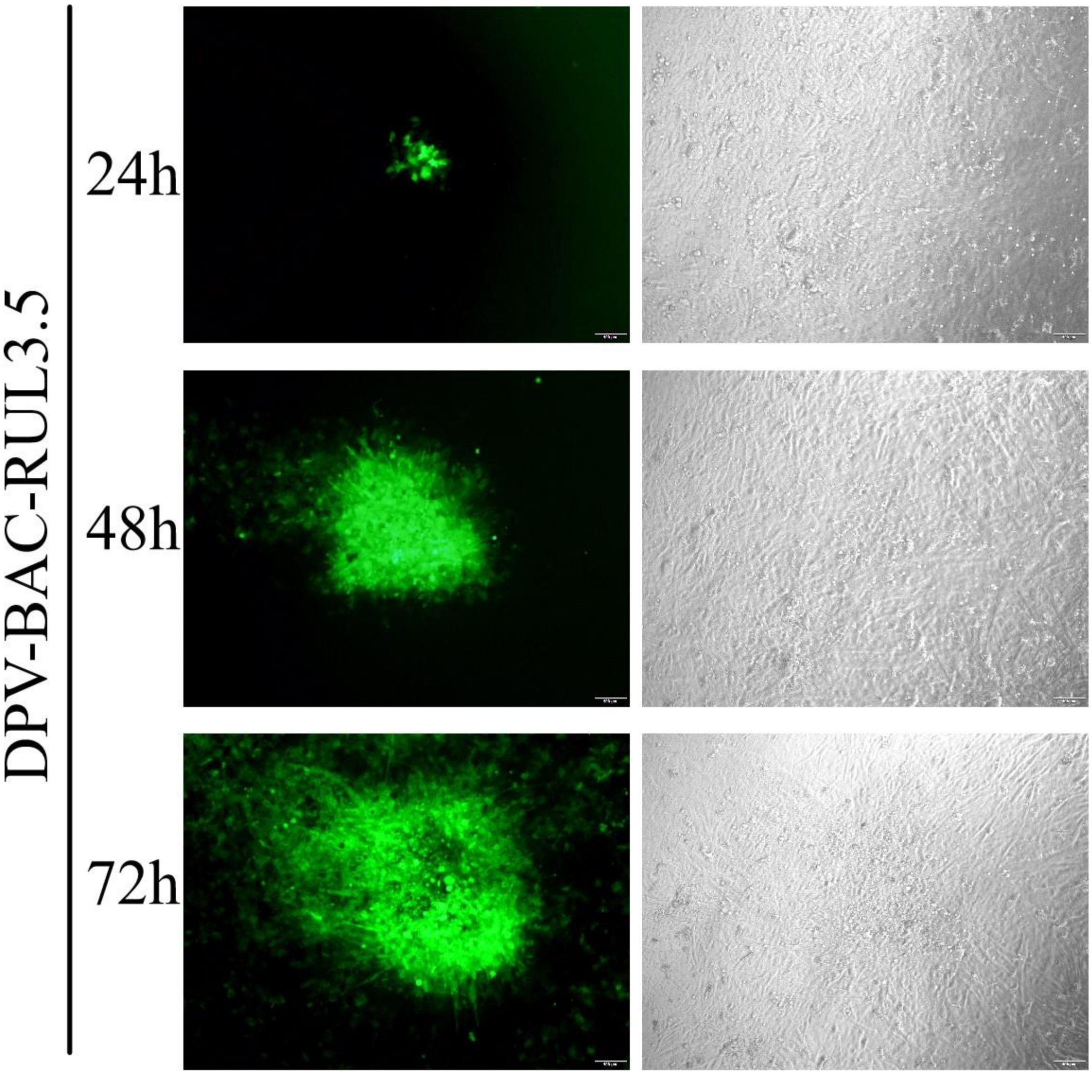

Figure 4. (**D**): Rescue of the DPV-BAC-RUL3.5 deletion mutant. The correctly sequenced positive infectious clonal plasmids were transfected into DEFs to observe the formation of green viral fluorescent plaques.

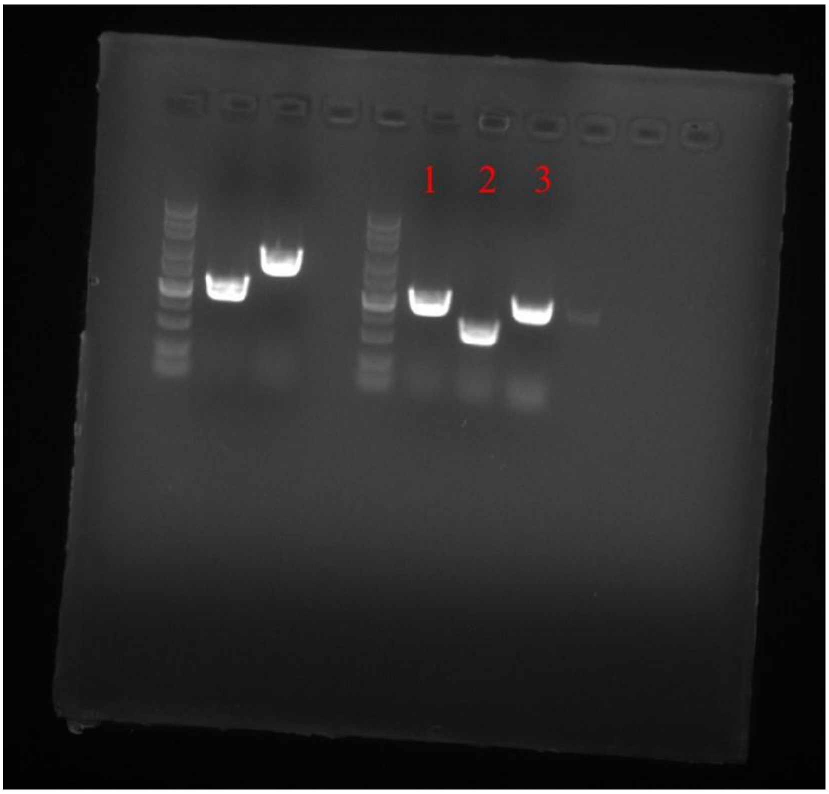

Figure 4. (**E**): PCR Identification of DPV-BAC-δUL3.5 and DPV-BAC-RUL3.5. Lane 1: Using DPV-BAC DNA as the template; Lane 2: Using DPV-BAC-δUL3.5 DNA as the template; Lane 3: Using DPV-BAC-RUL3.5 DNA as the template.

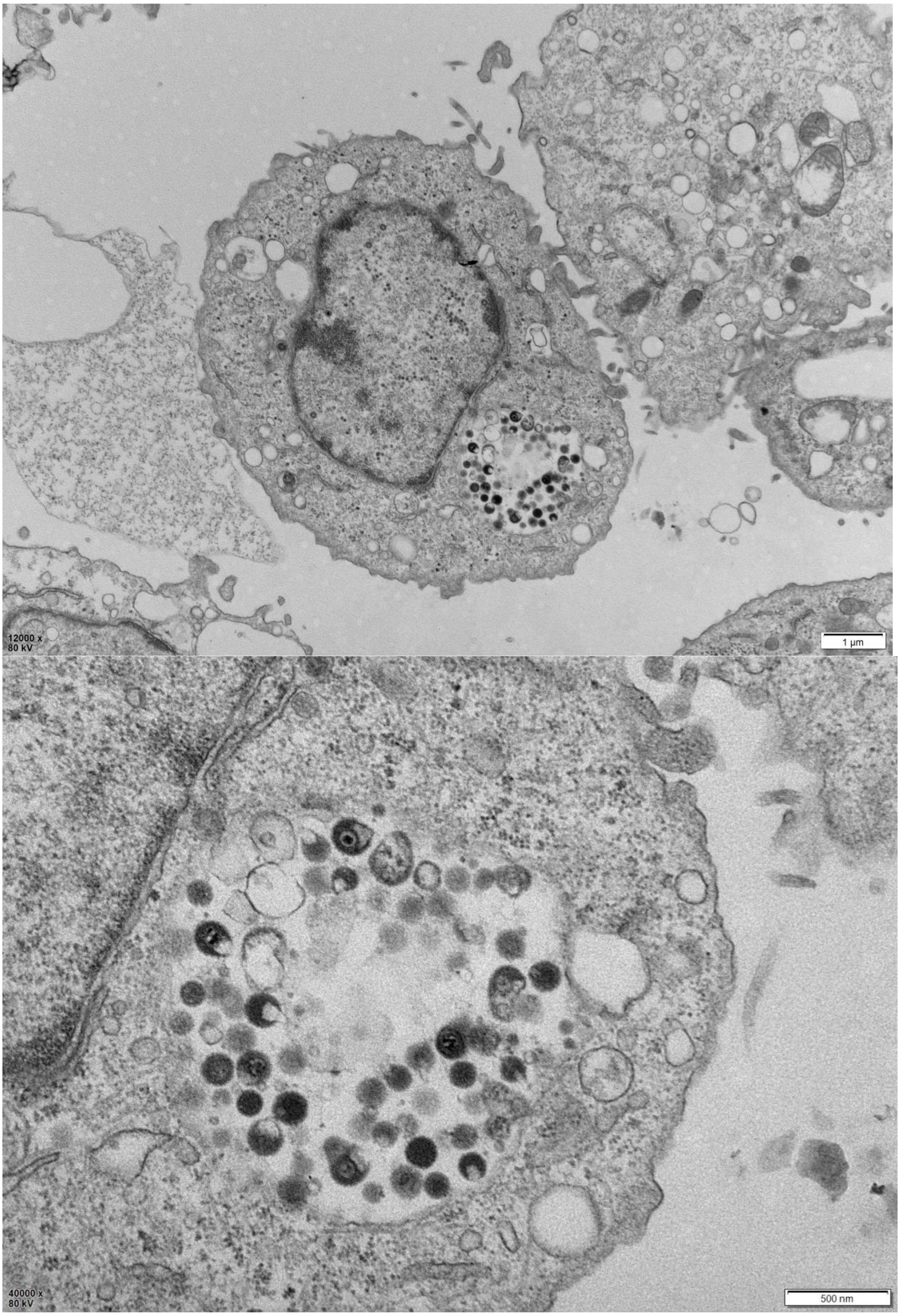

Figure 7. (**A-B**): Electron microscopy analysis of DPV-BAC.

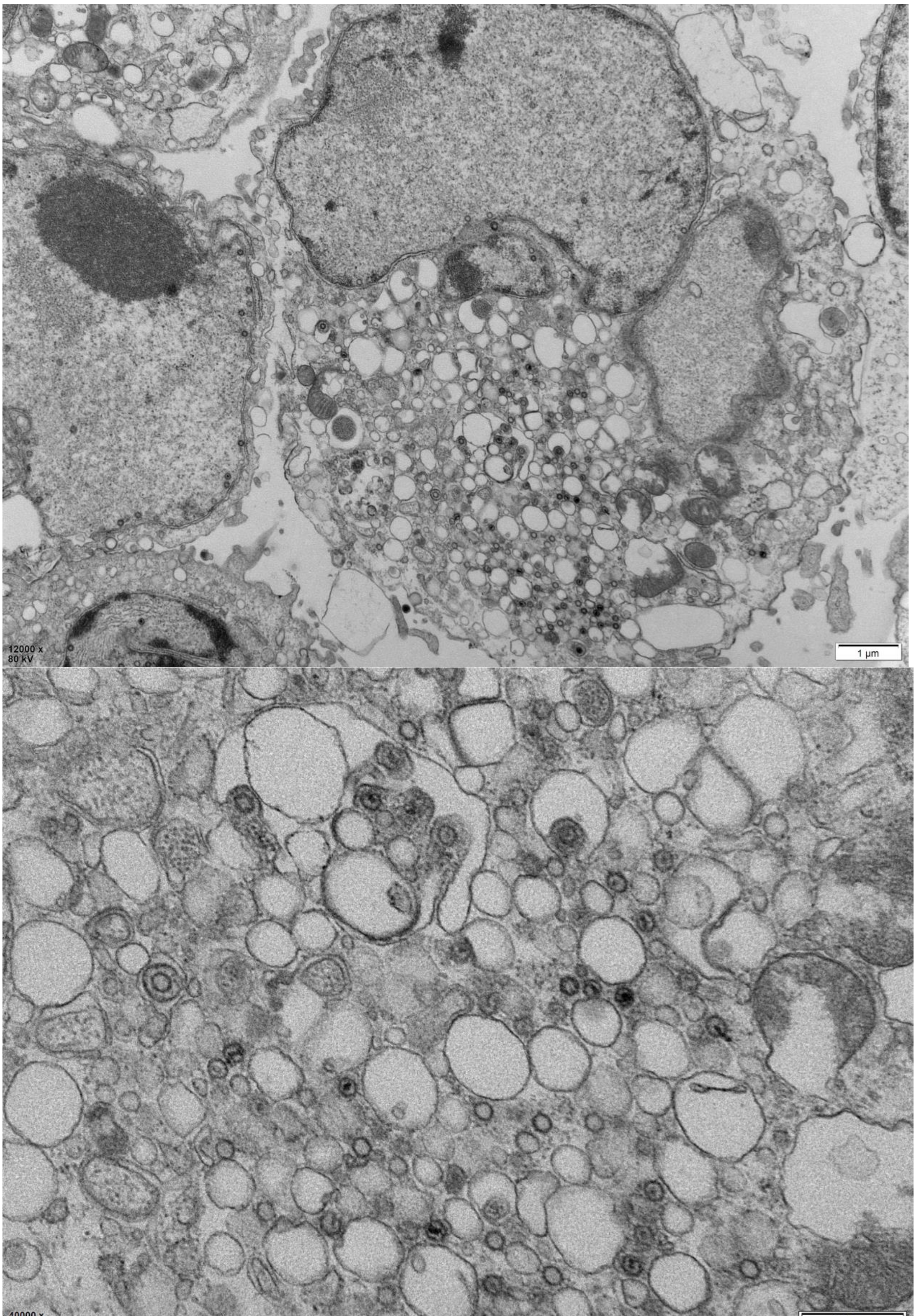

Figure 7. (**C-D**): Electron microscopy analysis of DPV-BAC-δUL3.5.

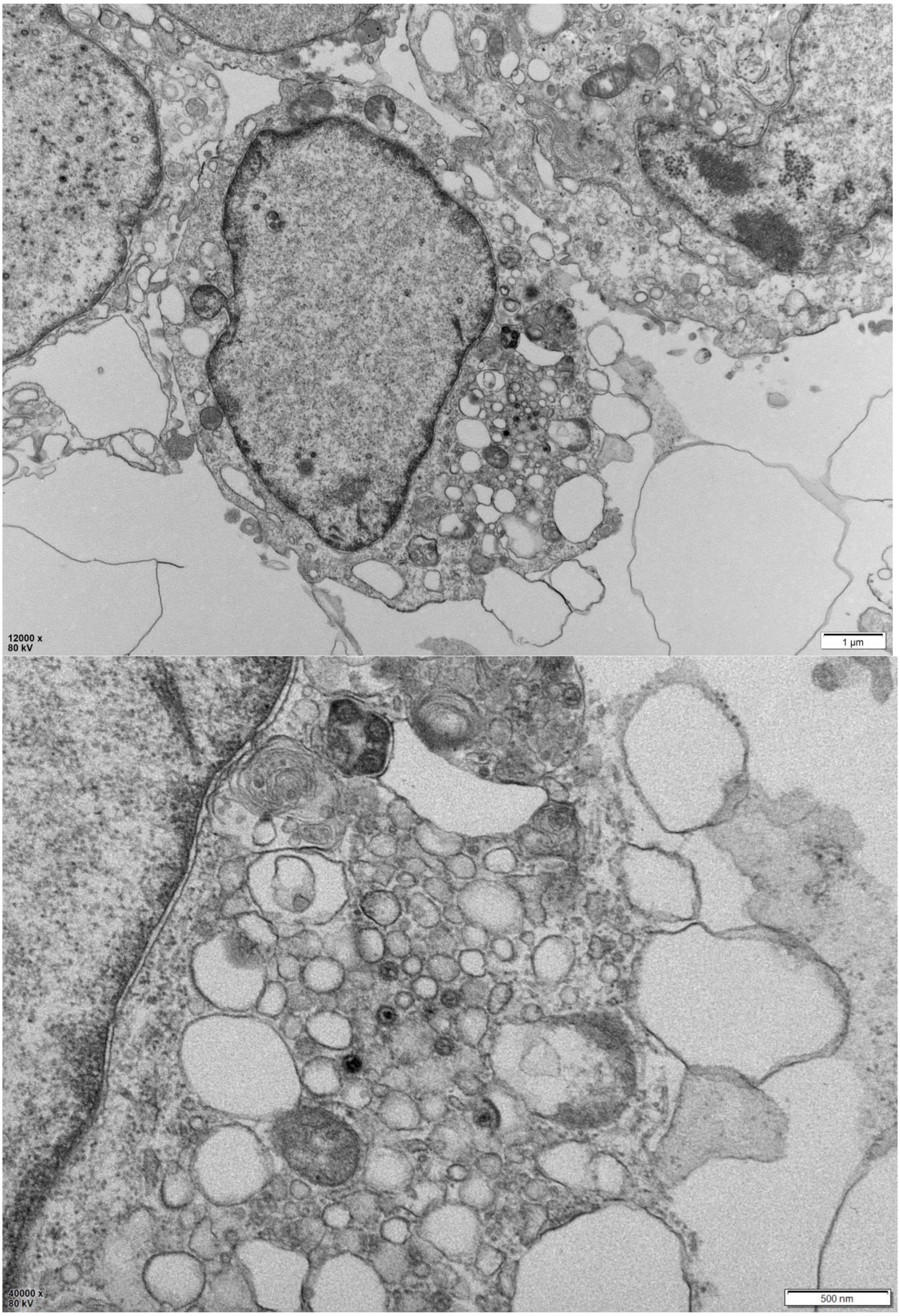

Figure 7. (**E-F**): Electron microscopy analysis of DPV-BAC-RUL3.5.

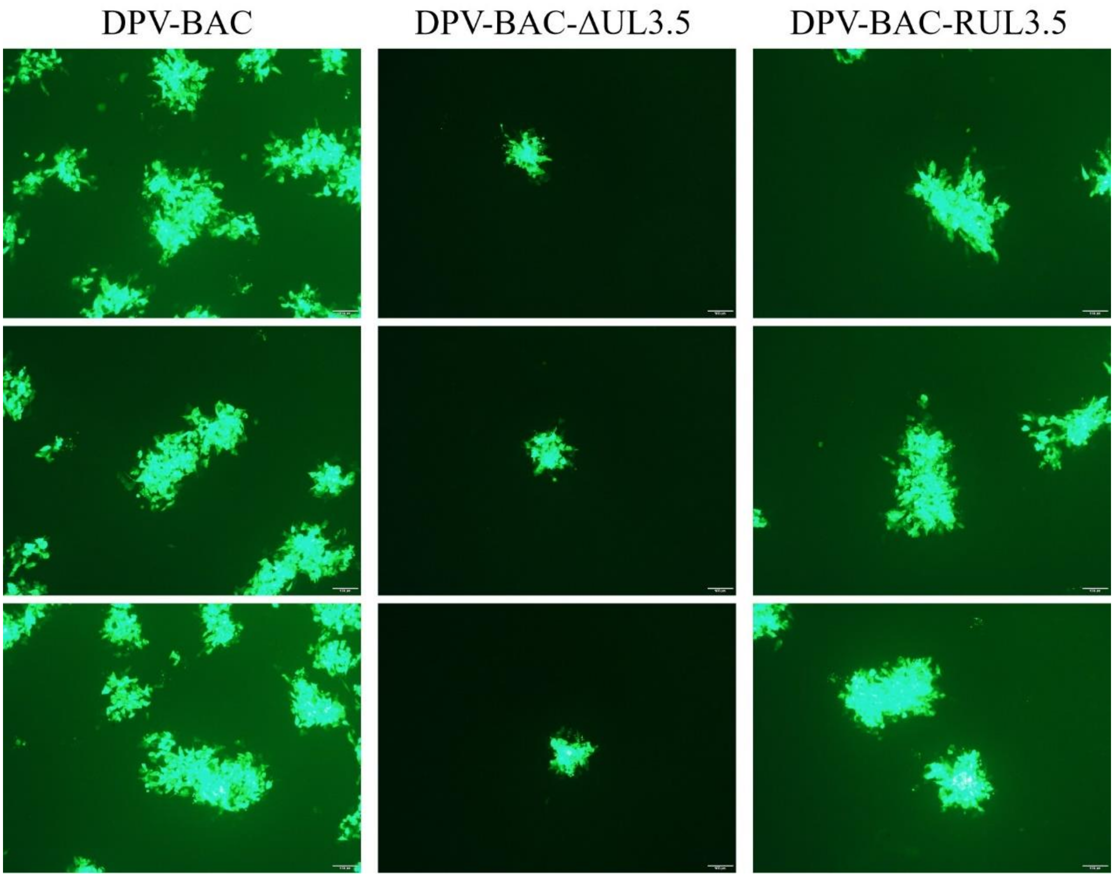

Figure 8. Impact of UL3.5 on DPV cell-to-cell spread. (A): Viral fluorescent plaques produced after infection of cells with the three virus strains.
